# Convergent targeting of FUNDC1‐dependent mitophagy sensitises and overcomes resistance to EGFR inhibition

**DOI:** 10.1002/ctm2.70685

**Published:** 2026-05-18

**Authors:** Fan Xu, Xiaoshan Wang, Min Li, Yi Li, Xiaojuan Li, Qingqing Yan, Fanming Kong, Qihong Huang, Xin Cao, Ying Xue

**Affiliations:** ^1^ Cancer Center Zhongshan Hospital Fudan University Shanghai China; ^2^ Institute of Clinical Sciences Zhongshan Hospital Fudan University Shanghai China; ^3^ Department of Oncology First Teaching Hospital of Tianjin University of Traditional Chinese Medicine Tianjin China; ^4^ National Clinical Research Center for Chinese Medicine Tianjin China; ^5^ Department of Central Laboratory Shandong Provincial Hospital Affiliated to Shandong First Medical University Jinan China; ^6^ Engineering Laboratory of Urinary Organ and Functional Reconstruction of Shandong Province Shandong Provincial Hospital Affiliated to Shandong First Medical University Jinan China; ^7^ The First Affiliated Hospital of Zhejiang Chinese Medical University Hangzhou China; ^8^ State Key Laboratory of Cardiovascular Disease Fuwai Hospital National Center for Cardiovascular Disease Chinese Academy of Medical Sciences and Peking Union Medical College Beijing China; ^9^ Tianjin Cancer Institute of Traditional Chinese Medicine Tianjin China; ^10^ Department of Oncology Shandong Provincial Hospital Affiliated to Shandong First Medical University Shandong China

**Keywords:** ABCB6, EGFR‐mutant NSCLC, FUNDC1‐mediated mitophagy, mitochondrial dysfunction, nitidine, osimertinib resistance

## Abstract

Combination therapies are critical for enhancing and prolonging the efficacy of EGFR inhibitors. Here, we uncover FUNDC1‐dependent mitophagy as a key protective mechanism in EGFR‐mutant non‐small cell lung cancer (NSCLC). We discover that nitidine, a bioactive component of the traditional Xihuang Pill formulation, synergises with the EGFR inhibitor osimertinib. Mechanistically, nitidine and osimertinib synergistically disrupt FUNDC1‐mediated mitophagy, leading to mitochondrial dysfunction and accumulation of reactive oxygen species in EGFR‐mutant NSCLC. We further show that both osimertinib and nitidine decrease HIF‐1α protein levels, thereby downregulating FUNDC1 expression. Nitidine‐induced downregulation of HIF‐1α and FUNDC1 depends on the mitochondrial transporter ABCB6. Notably, acquired resistance to osimertinib exhibits adaptive downregulation of FUNDC1, rendering resistant EGFR‐mutant NSCLC cells more sensitive to nitidine. Collectively, these findings position nitidine as a promising therapeutic strategy to enhance the efficacy of EGFR inhibitors and overcome osimertinib resistance in EGFR‐mutant NSCLC.

## Key points

- Nitidine synergises with osimertinib in EGFR‐mutant NSCLC.
- Nitidine and osimertinib converge on HIF‐1α/FUNDC1‐mediated mitophagy.
- ABCB6 is required for the nitidine‐associated HIF‐1α/FUNDC1 response.
- Adaptive FUNDC1 downregulation sensitises osimertinib‐resistant cells to nitidine monotherapy.

## Graphical abstract

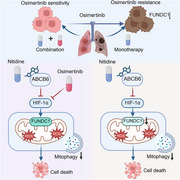

## 1 INTRODUCTION

The identification of epidermal growth factor receptor (EGFR) mutations as key oncogenic drivers in non–small cell lung cancer (NSCLC) has fundamentally transformed therapeutic approaches and paved the way for the development of EGFR tyrosine kinase inhibitors (TKIs).^1^ Osimertinib (Osi, AZD9291), a third‐generation EGFR‐TKI, has shown marked efficacy in tumours carrying the EGFR T790M mutation, which commonly arises as a mechanism of resistance to first‐generation EGFR‐TKIs such as gefitinib and erlotinib.^2^, ^3^, ^4^, ^5^, ^6^, ^7^ By selectively targeting mutant EGFR variants, including L858R/T790M and exon 19 deletions, Osi provides substantial survival benefits for patients with advanced NSCLC. However, acquired resistance to Osi remains a major clinical challenge, with resistance mechanisms being highly heterogeneous.^8^, ^9^, ^10^ Secondary EGFR mutations (e.g., C797X, L718X and G724X) have been identified but account for only a minority (10%–26%) of resistant cases, suggesting the involvement of additional resistance drivers.^11^, ^12^, ^13^ Notably, acquired resistance arises not only through the selection of pre‐existing resistant clones but also via the emergence of drug‐tolerant persister cells (DTPCs), which survive initial treatment through adaptive, non‐mutational mechanisms.^14^, ^15^, ^16^, ^17^, ^18^, ^19^, ^20^, ^21^ Over time, DTPs may acquire stable resistance through both mutational and non‐mutational alterations, leading to therapeutic failure. Recent clinical studies have further expanded the use of Osi, but have also underscored that treatment adaptation and acquired resistance remain major barriers to durable disease control. This highlights the limitation of focusing solely on EGFR inhibition and underscores the need for strategies that go beyond targeting EGFR mutations alone. Therefore, the development of effective combination therapies is urgently required and could have a significant impact on improving treatment outcomes for EGFR‐mutant NSCLC.^22^ While many combination strategies have focused on bypass signalling or resistance‐associated genomic alterations, the role of mitochondrial quality control, particularly mitophagy, in Osi response and resistance remains much less well defined.^23^

In parallel with targeted therapies, traditional Chinese medicine (TCM) has gained increasing recognition as a complementary approach in oncology. Xihuang Pill (XHP), an approved classical TCM formula (state medical permit number Z11020073), has been widely used in China as an adjuvant cancer treatment. Traditionally, it was prescribed for conditions such as carbuncles, deep abscesses and scrofula, while its modern clinical applications now extend to various cancers.^24^, ^25^ XHP inhibits cancer cell proliferation and reprogrammes the tumour immune microenvironment by regulating key molecules involved in immunogenic cell death, including HSP70, ATP and HMGB1, through pathways such as PI3K/AKT/NF‐κB, particularly in breast cancer.^26^, ^27^ Clinically, when combined with chemotherapy or radiotherapy, XHP has demonstrated potential to prolong survival, improve response rates, enhance quality of life, regulate immune function, and reduce treatment‐related toxicities across multiple cancer types, including breast, colorectal, oesophagal, gastric and lung cancers.^28^ However, how XHP restrains lung cancer growth remains unclear.

In this study, we explored a combinatorial therapeutic vulnerability in EGFR‐mutant NSCLC and elucidated the mechanism underlying the synergy between XHP, its active component nitidine, and Osi. We found that nitidine and Osi converge on HIF‐1α/FUNDC1‐mediated mitophagy, and that nitidine‐induced suppression of HIF‐1α and FUNDC1 depends on ABCB6. Together, nitidine and Osi synergistically blocked mitophagy, leading to potent cell death. Moreover, we found that adaptive downregulation of FUNDC1 in Osi‐resistant cells creates a vulnerability associated with increased sensitivity to Nitidine. These findings provide a mechanistic basis for combining traditional medicine with targeted therapy and support mitophagy inhibition as a potential strategy to enhance Osi efficacy and exploit a resistance‐associated vulnerability in EGFR‐mutant NSCLC.

## 2 MATERIALS AND METHODS

### 2.1 Preparation of drug‐containing serum

Male Sprague–Dawley rats were randomly divided into two groups, a drug‐serum group and a blank‐serum group (*n* = 6 per group). The rat dose was derived from the human‐equivalent dosage using a factor of 6.17. The adult human dose of XHP is 6 g per day (based on 60 kg bodyweight), which is equivalent to 617 mg/kg·per day for rats. To ensure sufficient efficacy, the rat dose was set at 1851 mg/kg·per day (1.851 g/kg·per day). XHP‐containing serum was prepared by dissolving the drug in 0.9% saline and administering it to rats daily by gavage for 7 days. Following the final treatment, anaesthetised rats underwent aseptic abdominal aortic blood collection. Serum was separated by centrifugation, heat‐inactivated at 56°C for 30 min, filtered, and stored at −80°C.

### 2.2 Screening of XHP drug‐containing serum concentrations

XHP concentrations were set at 0%, 5%, 10%, 15% and 20%, with three replicates for each concentration. For the 20% group, the drug‐containing serum was used directly, while lower concentrations were mixed with blank serum.

### 2.3 Cell lines

The NSCLC cell lines and HEK293T were obtained from the Cell Bank of the China Academy of Sciences (Shanghai, China). The Osi‐resistant cell lines PC9‐OR and NCI‐H1975‐OR were established by continuously exposing PC9 and NCI‐H1975 cells to increasing concentrations of Osi (up to 5 µM) over a 6‐month period. To maintain resistance, PC9‐OR and NCI‐H1975‐OR cells were cultured in the presence of Osi at appropriate concentrations.

### 2.4 UPLC‐Q/TOF‐MS analysis

Normal saline serum and XHP‐containing serum were freeze‐dried under vacuum, reconstituted in water/methanol (60:40), centrifuged at 16 000 × *g* for 20 min, and passed through a 0.22 µm filter. Samples were performed for untargeted component analysis. Compounds were separated on an ACQUITY UPLC HSS T3 column with a water/acetonitrile gradient in the presence of 0.1% formic acid. A SCIEX TripleTOF 6600 mass spectrometer with electrospray ionisation (ESI) was operated in data‐dependent acquisition (DDA) mode, collecting full‐scan and MS/MS spectra in both positive and negative polarities. Acquired data were processed with SCIEX OS software, and compounds were identified by matching against a dedicated Traditional Chinese Medicine MS/MS spectral library.

### 2.5 Patient‐derived organoids (PDOs) culture and drug assay

Fresh tumour tissues were obtained with written informed consent and processed within 2 h of collection. Specimens were minced into approximately 1–2 mm fragments and digested in advanced DMEM/F12 supplemented with collagenase II, dispase, DNase I and Y‐27632 at 37°C with gentle agitation until adequate dissociation was achieved. Cell suspensions were filtered through a 70 µm strainer and embedded in growth‐factor‐reduced Matrigel as domes in pre‐warmed culture plates. PDOs were maintained in organoid‐specific medium, with medium changed every 2–3 days. For drug testing, PDOs were dissociated into small clusters, re‐seeded in Matrigel, and allowed to recover for 48 h before treatment with the indicated compounds. Organoid growth was recorded every 2 days, and cell survival was evaluated using the CellTiter‐Lum 3D viability assay kit (Beyotime, China).

### 2.6 Transfection and lentivirus production

For lentivirus production, HEK293T cells were co‐transfected with the indicated plasmids and the packaging plasmids PMD2G/PSPAX using Lipofectamine 2000. Virus‐containing supernatants were harvested, centrifuged, filtered through 0.22 µm filters, and used for subsequent infection.

### 2.7 Western blotting

Whole‐cell lysates were collected, and the lysate concentration was quantified by BCA assay. Equivalent amounts were separated by SDS‐PAGE, transferred to membranes, and subjected to blocking prior to immunoblot analysis. Membranes were incubated overnight at 4°C with primary antibodies against FUNDC1, HIF‐1α, ABCB6, PINK1, BNIP3, BNIP3L/NIX, TOM20, TIM23, LC3A/B, SQSTM1/p62, BID, cleaved caspase‐3, GAPDH or β‐actin. After incubation with the appropriate secondary antibodies, immunoreactive bands were detected by ECL.

### 2.8 RT‐qPCR

Total RNA was isolated with TRIzol and reverse‐transcribed into cDNA using a commercial kit. qPCR was performed with SYBR Green on a real‐time PCR platform, and relative transcript levels were normalised to β‐actin using the 2^−ΔΔCt^ method. Primer sequences for FUNDC1, HIF1A, BNIP3, BNIP3L/NIX and mitochondrial genes for mtDNA/nDNA quantification are listed in Table S2.

### 2.9 Cell counting kit‐8 (CCK‐8) and colony formation assay

Cell viability was measured by cell counting kit‐8 (CCK‐8) assay (Vazyme, Nanjing, China). Cells were seeded in 96‐well plates at 5000 per well, allowed to attach overnight, and treated for 48 h. For colony formation assays, cells were cultured in 12‐well plates under the indicated treatments for 14 days with medium replacement every 3 days, followed by crystal violet staining and image acquisition.

### 2.10 Flow cytometry

Apoptotic fractions were measured using Annexin V‐FITC/PI staining performed with a commercial apoptosis assay kit (BestBio, China).

### 2.11 mt‐Keima flow cytometric analysis of mitophagy

For mt‐Keima assays, cells stably expressing mitochondrial‐targeted Keima were treated as indicated, harvested without fixation, and analysed by flow cytometry using dual excitation to distinguish neutral mitochondria from acidic mitolysosomes, as previously described.^29^ The proportion of cells with mitophagy events was quantified on the basis of the acidic mt‐Keima signal after exclusion of debris and doublets.

### 2.12 Mitophagy fluorescence

Mitophagy in live cells was assessed using a mitophagy detection kit (Dojindo, Japan) according to the manufacturer's instructions. Mitophagy fluorescence intensity was quantified from at least three independent fields per condition using ImageJ.

### 2.13 mtDNA/nDNA ratio

Mitochondrial content‐associated changes were further evaluated by quantitative PCR analysis of the mtDNA/nDNA ratio. mtDNA levels were quantified using mtCYTB, mtCO1 and mtATP6, and normalised to nuclear DNA RPL13A.^29^

### 2.14 JC‐1 staining

Mitochondrial membrane potential was assessed by JC‐1 staining (Beyotime Biotechnology). All samples were visualised by laser confocal microscopy (Leica, Germany).

### 2.15 Measurement of mitochondrial ROS (mROS)

Mitochondrial oxidative stress was measured by MitoSOX Red staining. Briefly, cells cultured in confocal dishes were exposed to 5 µM MitoSOX Red (YEASEN, Shanghai, China) for 10 min at 37°C under light‐protected conditions, washed three times with warm PBS, and then examined by confocal microscopy (Leica, Germany).

### 2.16 In vitro CRISPR screening

A pooled genome‐wide CRISPR knockout screen was conducted in PC‐9 cells using the human GeCKO v2 library (Addgene #73179). Cells were transduced at an MOI of approximately 0.3 in the presence of 8 µg/mL polybrene, selected with 1 µg/mL puromycin, and maintained at greater than 500‐fold sgRNA coverage. After recovery, cells were treated with 10 µM nitidine or DMSO control, and genomic DNA was extracted from each condition using a column‐based genomic DNA kit (Qiagen, #51304). sgRNA cassettes were amplified by two‐step PCR, subjected to next‐generation sequencing, and analysed using MAGeCK‐based pipelines to identify enriched and depleted genes. Genes with increased sgRNA abundance under nitidine treatment were interpreted as candidate resistance genes, whereas genes with decreased sgRNA abundance were considered candidate sensitisers.

### 2.17 Cellular thermal shift assays (CETSA)

CETSA was performed to assess nitidine binding to ABCB6. Cells were lysed by three freeze–thaw cycles in protease inhibitor‐containing PBS, and clarified lysates were incubated with DMSO or nitidine for 2 h at 37°C. For temperature‐gradient CETSA, aliquots were heated for 5 min at the indicated temperatures, rapidly cooled on ice, and centrifuged to separate soluble fractions. For concentration‐dependent CETSA, lysates were incubated with the indicated concentrations of nitidine and heated at 45°C before centrifugation. Soluble proteins were examined by Western blotting to evaluate ABCB6 stability.

### 2.18 In vivo study

PC‐9 xenografts were generated by subcutaneous injection of 1 × 10^6^ cells in 100 µL PBS into nude mice. Once the tumour burden approached 60 mm^3^, mice were allocated randomly to the indicated groups (*n* = 6 per group). XHP treatment consisted of daily oral gavage at 2.16 g/kg formulated in 0.5% CMC‐Na, whereas Osi was administered orally at 25 mg/kg using a 5‐on/2‐off schedule. Nitidine (8 mg/kg once every other day) were administered by intraperitoneal injection; the nitidine regimen was selected on the basis of pilot efficacy and tolerability testing. Tumour volume and weight were monitored every 2–3 days, with volume calculated as *V* = [Length × (Width^2^)]/2.

For in vivo rescue experiments, xenografts were established in the same manner using PC‐9 cells with the indicated treatments, including ABCB6 knockdown, HIF1A overexpression, FUNDC1 overexpression, or BID knockdown. Once tumours reached approximately 60 mm^3^, mice were treated with nitidine using the same dosing regimen as above, and tumour growth was monitored as described for the efficacy studies. At the experimental endpoint, tumours and major organs were collected for downstream analyses, including immunoblotting, serum biochemistry and histopathology.

### 2.19 Statistical analysis

Data from at least three independent experiments are presented as mean ± SEM. Statistical significance between two groups was assessed by an unpaired two‐tailed Student's *t*‐test. Group comparisons involving a single variable were analysed by one‐way ANOVA, followed by Tukey's multiple‐comparisons test, while those involving two variables were assessed by two‐way ANOVA with Tukey's correction. Significance was assigned at *p* < .05; ns indicates not significant; *p < .05, **p < .01, ***p < .001.

## 3 RESULTS

### 3.1 XHP demonstrates selective efficacy and synergises with osimertinib in EGFR‐mutant NSCLC

**FIGURE 1:**
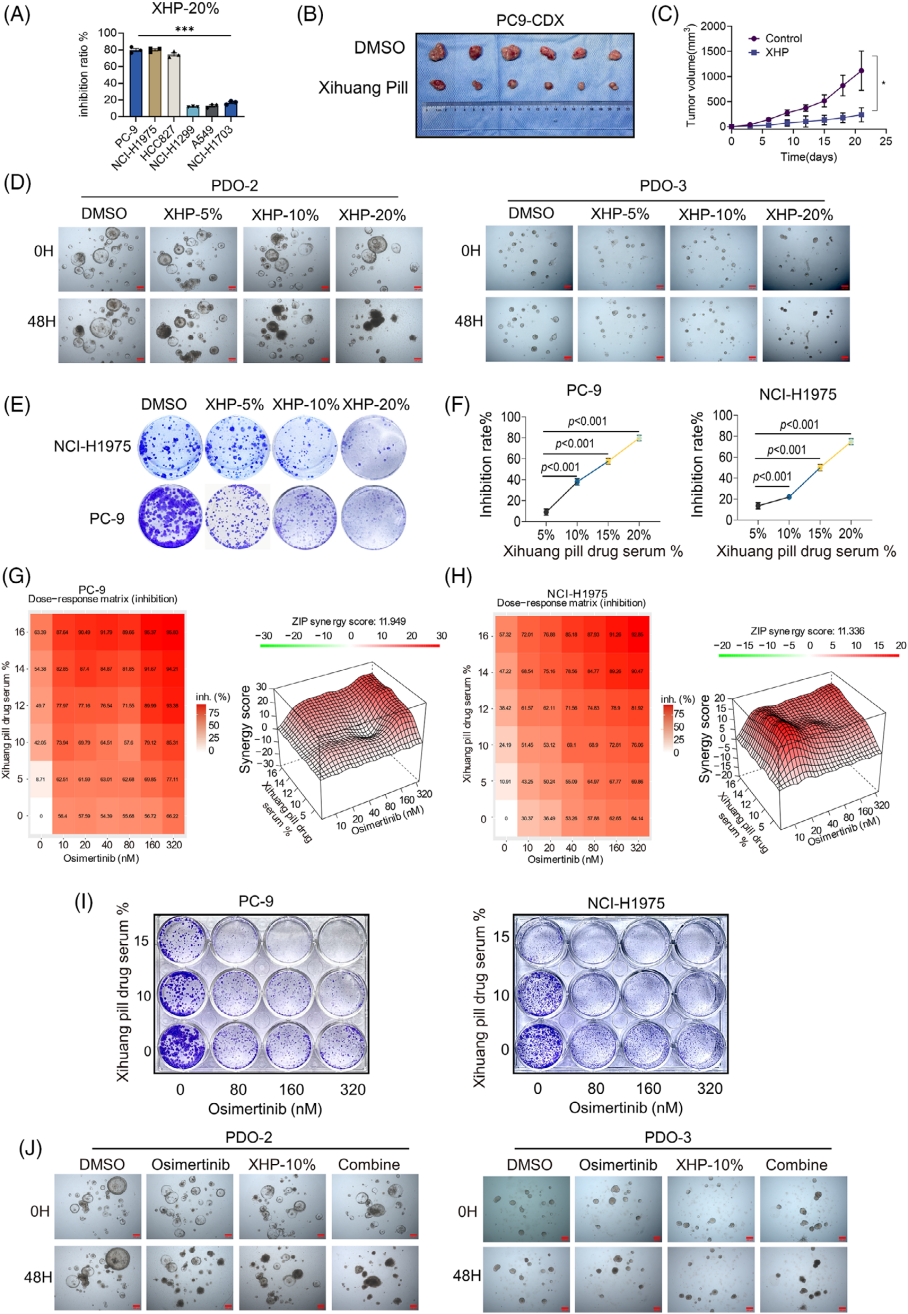
Xihuang Pill demonstrates anti‐tumour effects in EGFR‐mutant NSCLC, and enhances the efficacy of osimertinib. (A) Different NSCLC cell lines were treated with 20% concentrations of Xihuang Pill (XHP) for 48 h and subjected to CCK‐8 assays. Inhibition efficacy was assessed in both EGFR wildtype and mutant cell lines. (B and C) PC‐9 cells were subcutaneously injected into nude mice, and XHP or vehicle controls were administered via oral gavage. Tumour growth was monitored in both groups. (B) Images of subcutaneous xenografts with or without XHP treatment. (C) Tumor volume measurements in nude mice following XHP treatment. (D) EGFR‐mutant NSCLC patient‐derived organoids (PDOs) were treated with varying concentrations of XHP (5%, 10%, 20%); scale bars = 200 µm. (E) Clonogenic assay of PC‐9 and NCI‐H1975 cells following treatment with DMSO or varying concentrations of XHP (5%, 10%, 20%). (F) Inhibition efficacy of PC‐9 and NCI‐H1975 cells following treatment with varying concentrations of XHP (5%, 10%, 15%, 20%). (G and H) Synergy analysis of XHP and osimertinib (Osi) combination treatment in PC‐9 (G) and NCI‐H1975 (H) cells. Left: Dose–response matrix showing inhibition efficacy of XHP and Osi. Right: 3D synergy distribution and synergy score based on the ZIP model, with scores greater than 10 indicating synergy. (I) Clonogenic assay of PC‐9 and NCI‐H1975 cells following treatment with XHP and Osi. (J) EGFR‐mutant NSCLC PDOs treated with Osi, XHP, or the combination of XHP and Osi. Scale bars = 200 µm. Data are presented as mean ± SEM. Statistical analyses are determined by two‐tailed unpaired Student's *t*‐test (A and C); one‐way ANOVA with Tukey's multiple‐comparisons test (F). ***p < .001.

Given its robust efficacy in various cancers, we assessed the potential of XHP in combination therapy for lung cancer. Our results showed that XHP, when used as an adjuvant treatment, significantly enhanced tumour response rates and slowed disease progression in lung cancer patients, demonstrating particularly superior efficacy in NSCLC (Figure S1). Furthermore, compared with EGFR‐wildtype NSCLC cell lines, EGFR‐mutant NSCLC cell lines showed a higher inhibition rate after XHP treatment (Figure 1A). In an EGFR‐mutant PC‐9 xenograft model, XHP treatment markedly suppressed tumour growth and reduced tumour volume (Figure 1B,C). These data suggest that XHP exerts preferential anti‐tumour efficacy in EGFR‐mutant NSCLC. To further assess its clinical relevance, we developed EGFR‐mutant NSCLC patient‐derived organoids (PDOs) and treated them with XHP. Notably, XHP alone exhibited potent growth‐inhibitory effects across all PDO models (Figure 1D; Figure S2A). Having established XHP's selectivity, we examined its effects in EGFR‐mutant PC‐9 and NCI‐H1975 cells. XHP dose‐dependently inhibited cell proliferation and clonogenic growth (Figure 1E,F; Figure S2B). Osimertinib (Osi), a third‐generation EGFR tyrosine kinase inhibitor, is the first‐line standard of care for EGFR‐mutant NSCLC, but durable disease control with monotherapy remains limited. Given the selective activity of XHP in EGFR‐mutant models and its distinct mechanism of action, we investigated whether XHP could enhance Osi efficacy. Using the Zero Interaction Potency (ZIP) model, we observed strong synergy between XHP and Osi in PC‐9 (ZIP score = 11.95) and NCI‐H1975 cells (ZIP score = 11.34), with ZIP scores greater than 10 indicating synergy (Figure 1G,H). The combination nearly abolished clonogenic growth (Figure 1I; Figure S2C). Consistently, in EGFR‐mutant PDOs, a 48‐h XHP–Osi treatment produced greater tumour growth inhibition than either agent alone (Figure 1J; Figure S2D), further supporting its translational potential.

### 3.2 Nitidine is the key active component driving the anti‐tumour activity of XHP against EGFR‐mutant NSCLC

**FIGURE 2:**
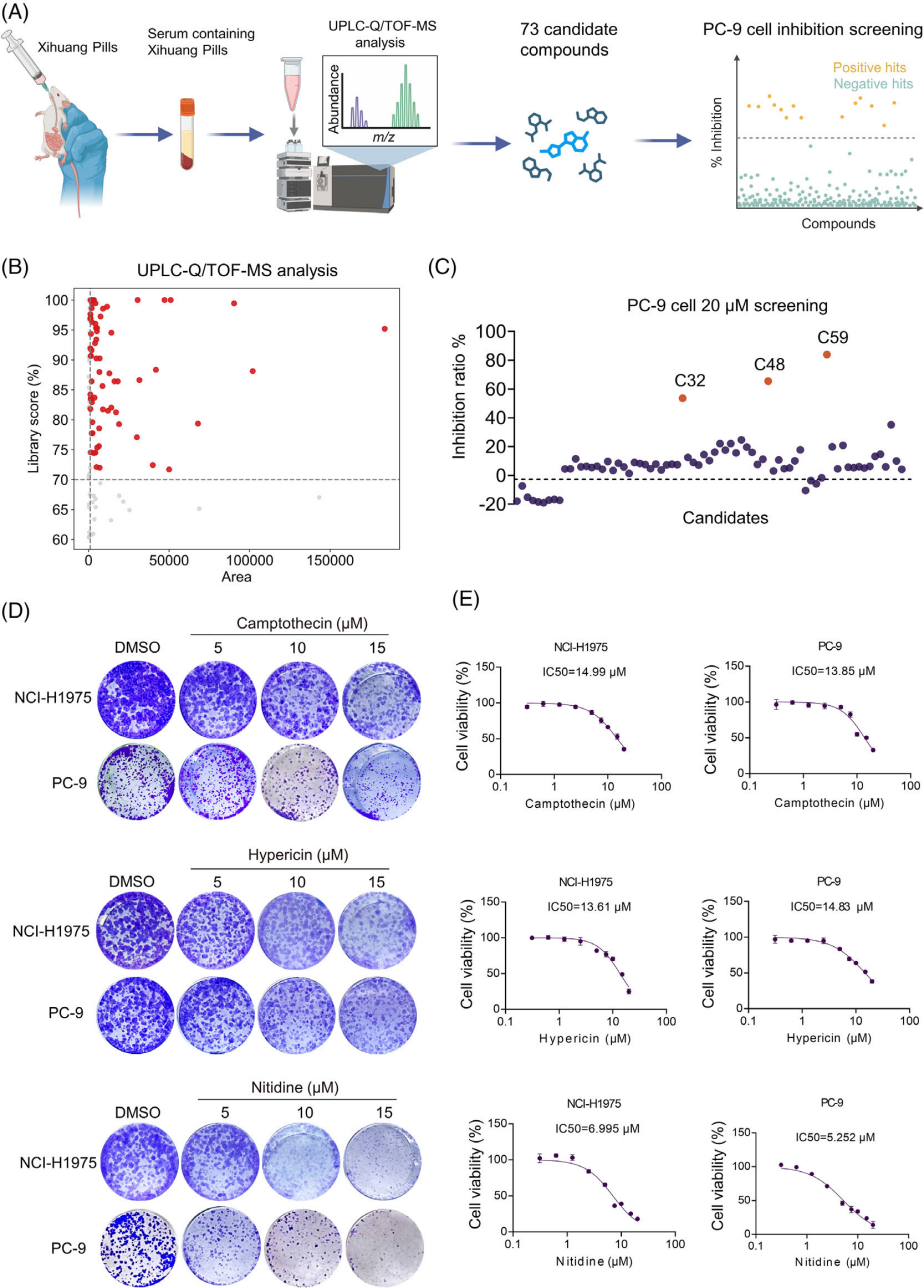
Identification of nitidine as a key bioactive component of Xihuang Pill (XHP). (A) Schematic of UHPLC‐HRMS analysis to identify active components of XHP. Briefly, powdered XHP was administered to SD rats via gavage to obtain drug‐containing serum, followed by mass spectrometry analysis to identify its constituents. A total of 73 potential candidates were selected and tested for their activity in cell viability assays using PC‐9 cells. (B) UPLC‐Q/TOF‐MS analysis of XHP related components detected in plasma from SD rats. The scatter plot shows the relationship between peak area and library matching score. The horizontal axis represents peak area, and the vertical axis represents library score (%). The dashed line indicates the screening threshold of 70%; red dots represent features with library scores above the threshold, while gray dots reprsent features below the threshold. (C) Inhibition efficacy of PC‐9 cells following treatment with the selected 73 compounds. (D) Clonogenic assay of PC‐9 and NCI‐H1975 cells treated with varying concentrations of camptothecin (C32), hypericin (C48) or nitidine (C59). (E) Inhibition efficacy of PC‐9 and NCI‐H1975 cells following treatment with DMSO or varying concentrations of camptothecin, hypericin or nitidine.

Given its promising efficacy against EGFR‐mutant NSCLC, we aimed to identify the key bioactive constituents of XHP. To achieve this, we conducted UHPLC‐HRMS analysis of drug‐containing serum collected from Sprague–Dawley rats, thereby elucidating its chemical composition. Seventy‐three components with an area greater than 2000 and a library score greater than 70% (Figure 2A,B; Table S1) were selected for further viability assays in PC‐9 cells. Among these, three monomeric compounds—camptothecin (C32), hypericin (C48) and nitidine (C59)—demonstrated potent cytotoxicity in PC‐9 cells, with nitidine showing the highest efficacy (Figure 2C). We then performed dose–response viability and colony‐formation assays in EGFR‐mutant NSCLC cell lines (NCI‐H1975 and PC‐9), revealing that nitidine was the most potent of the three, exhibiting the lowest IC50 and the strongest suppression of clonogenic growth (Figure 2D,E). We further evaluated the interactions of nitidine with camptothecin and hypericin, and found that neither combination showed clear synergy, with all ZIP scores below 10, indicating predominantly additive effects (Figure S3A,B). In addition, analyses of normal‐cell viability, serum biochemistry and histopathology showed that nitidine had a measurable in vitro selectivity window and acceptable tolerability in vivo (Figure S4A–D).

**FIGURE 3:**
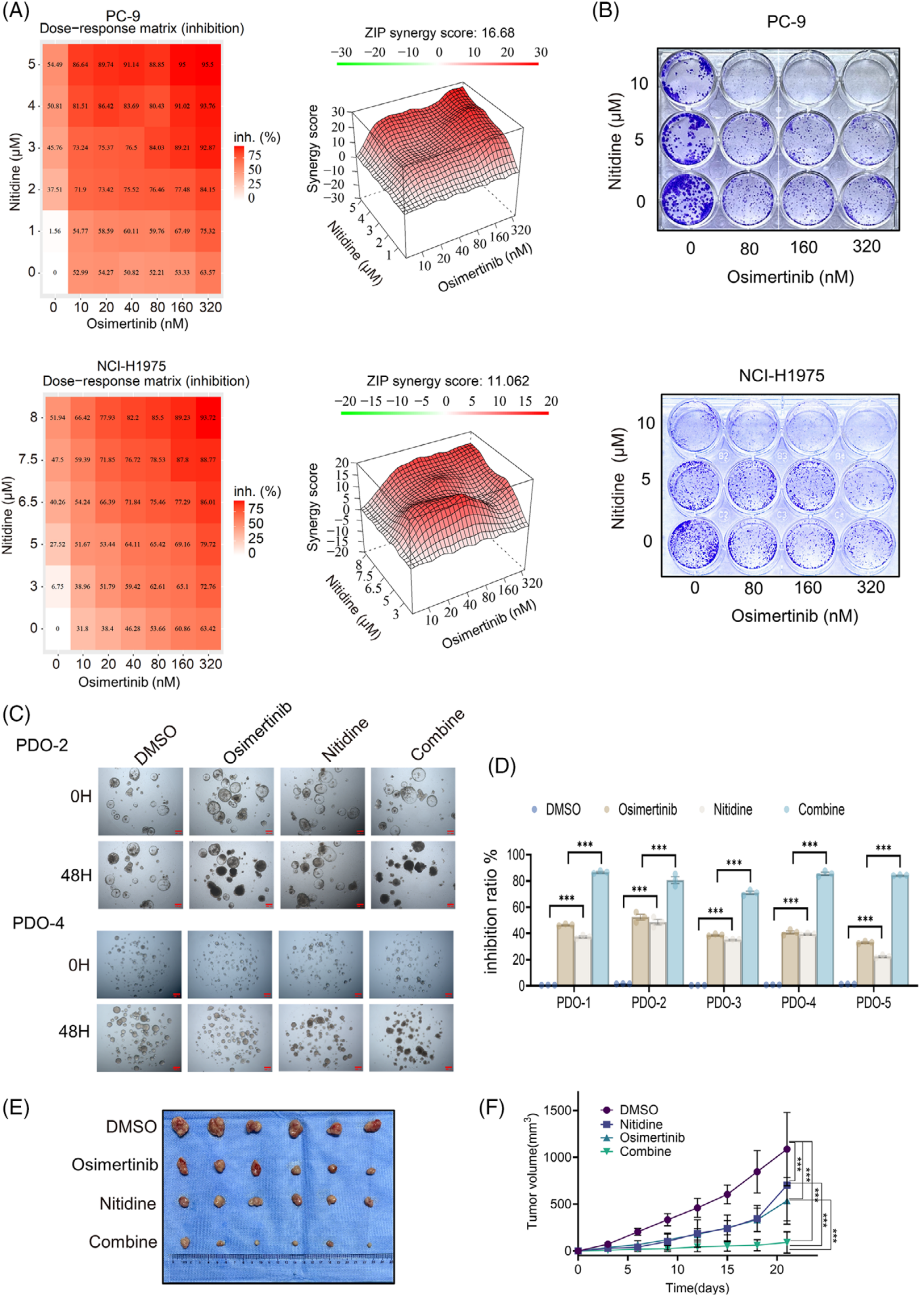
Nitidine synergises with osimertinib in EGFR‐mutant NSCLC. (A) Synergy analysis of the nitidine and Osi combination in PC‐9 (top panel) and NCI‐H1975 (bottom panel) cells. Left: Dose–response matrix showing inhibition efficacy of nitidine and Osi. Right: 3D synergy distribution and synergy scores for nitidine and Osi based on the ZIP model. (B) Clonogenic assay of PC‐9 and NCI‐H1975 cells following treatment with a combination of Osi and nitidine. (C) EGFR‐mutant NSCLC patient‐derived organoids (PDOs) treated with Osi (100 nM), nitidine (10 µM), or the combination of nitidine and Osi; scale bar = 200 µm. (D) Inhibition efficacy of EGFR‐mutant NSCLC PDOs following treatment with Osi, nitidine, or the combination of nitidine and Osi. (E and F) PC‐9 cells were subcutaneously injected into nude mice, and Osi was administered via oral gavage while nitidine was administered by intraperitoneal injection. Tumour growth was measured in the treatment groups. (E) Images of tumours in subcutaneous xenografts treated with nitidine, Osi, or the combination regimen. (F) Tumour volume measurements in nude mice following treatment with nitidine, Osi, or the combination. Data are presented as mean ± SEM. Statistical analyses were determined by two‐way ANOVA with Tukey's multiple‐comparisons test (D); one‐way ANOVA with Tukey's multiple‐comparisons test (F). ***p < .001.

We next assessed the synergistic interaction between nitidine and Osi in PC‐9 and NCI‐H1975 cells. The combination exhibited strong synergy, with ZIP scores of 16.68 and 11.06, respectively, and almost completely suppressed clonogenic growth (Figure 3A,B). We then evaluated the nitidine–Osi combination in EGFR‐mutant PDOs and PC‐9 xenografts. Across PDO models, the combination consistently induced stronger growth inhibition than either single agent (Figure 3C,D; Figure S5A–C), and in PC‐9 xenografts, it achieved greater tumour suppression than monotherapies (Figure 3E,F), demonstrating superior anti‐tumour activity in EGFR‐mutant NSCLC.

### 3.3 Nitidine synergises with osimertinib by inhibiting FUNDC1‐mediated mitophagy in EGFR‐mutant NSCLC

**FIGURE 4:**
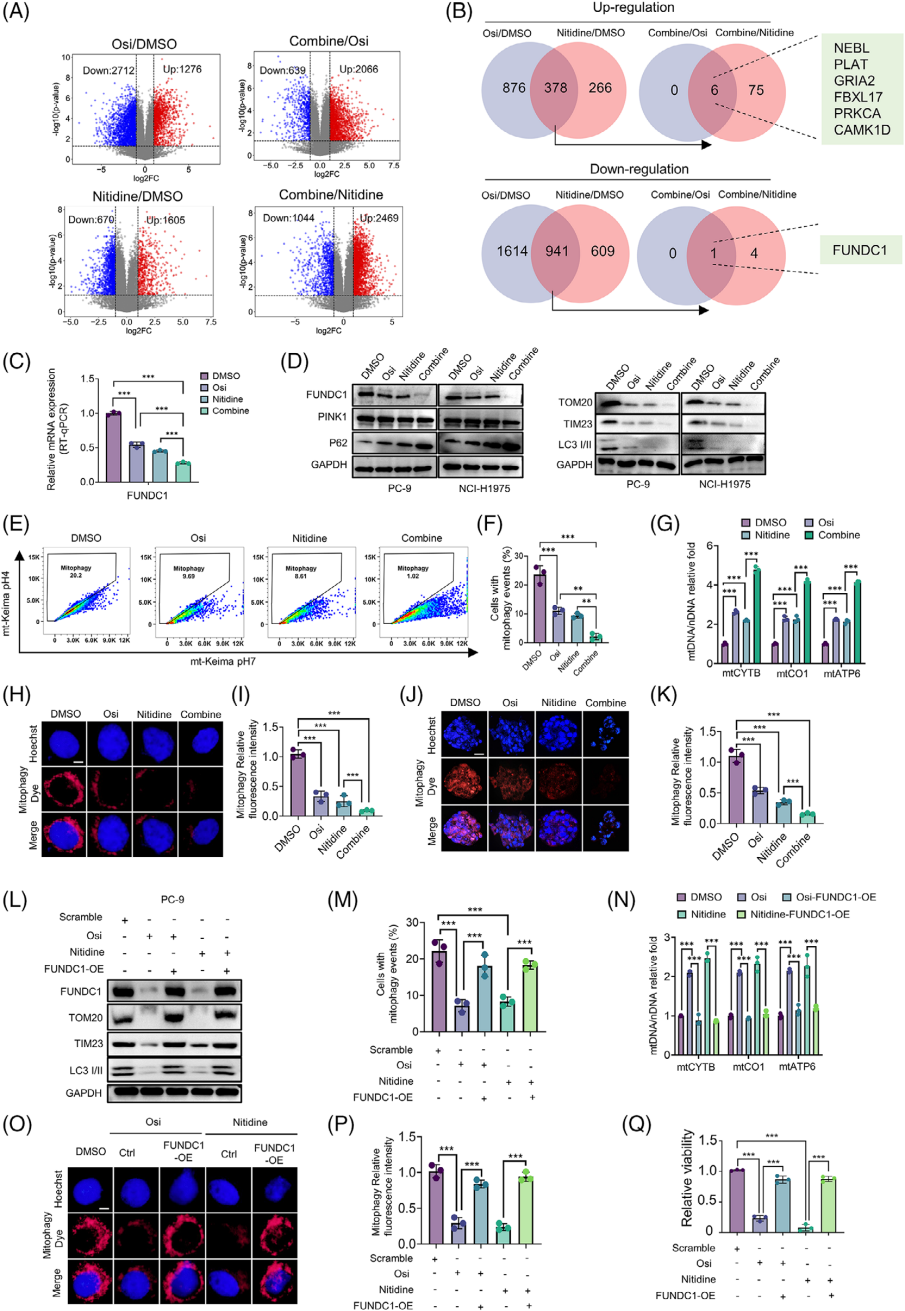
Blockade of FUNDC1‐mediated mitophagy is essential for the synergistic activity of nitidine and osimertinib in EGFR‐mutant NSCLC. (A and B) RNA sequencing was performed on PC‐9 cells treated with DMSO, nitidine (10 µM), osimertinib (Osi) (100 nM), or their combination for 48 h. (A) Volcano plots show the differentially expressed genes. (B) Venn diagrams illustrate the overlap of differentially expressed genes across the treatments. (C) RT‐qPCR for FUNDC1 mRNA in PC‐9 cell lines following treatment with nitidine, Osi, or the combination regimen. (D) Immunoblot analysis of FUNDC1, PINK1 and p62 (left), and TOM20, TIM23 and LC3‐I/II (right), in PC‐9 and NCI‐H1975 cells following treatment with nitidine, Osi, or the combination regimen. (E and F) mt‐Keima reporter for flow cytometry analysis. (E) mt‐Keima flow cytometry plots showing mitophagy in PC‐9‐mt‐Keima cells following treatment with nitidine, Osi, or the combination. (F) Quantification of the percentage of cells with mitophagy events in PC‐9‐mt‐Keima cells following treatments. (G) RT‐qPCR‐based quantification of the mtDNA/nDNA ratio in PC‐9 cells following treatment with nitidine, Osi, or the combination, as an indicator of mitochondrial mass/mitophagy‐associated changes. (H) Fluorescence‐based quantification of relative mitophagy signal intensity in PC‐9 cells following treatment with nitidine, osimertinib, or the combination; scale bar = 5 µm. (I) Quantification of the relative mitophagy fluorescence intensity in PC‐9 cells following treatments with nitidine, Osi, or the combination regimen. (J) Mitophagy was assessed by fluorescence microscopy in EGFR‐mutant NSCLC PDOs following treatment with nitidine, Osi, or the combination regimen; scale bar = 25 µm. (K) Quantification of the relative mitophagy fluorescence intensity in EGFR‐mutant NSCLC PDOs following treatments with nitidine, Osi, or the combination regimen. (L) Immunoblot analysis of FUNDC1, TOM20, TIM23 and LC3‐I/II in PC‐9 cells transduced with control vector or FUNDC1 overexpression (FUNDC1‐OE) and treated as indicated. (M) Quantification of the percentage of cells with mitophagy events in PC‐9‐mt‐Keima cells transduced with control vector or FUNDC1‐OE after treatment with Osi or nitidine. (N) RT‐qPCR‐based quantification of the mtDNA/nDNA ratio in PC‐9 cells transduced with control vector or FUNDC1‐OE after treatment with Osi or nitidine. (O) Representative fluorescence images showing mitophagy dye signal in control and FUNDC1‐overexpressing PC‐9 cells after treatment with Osi or nitidine. (P) Quantification of the relative mitophagy fluorescence intensity in control and FUNDC1‐overexpressing PC‐9 cells after treatment with osimertinib or nitidine. (Q) Relative cell viability of control and FUNDC1‐overexpressing PC‐9 cells after treatment with Osi or nitidine. Data are presented as mean ± SEM. Statistical analyses were determined by one‐way ANOVA with Tukey's multiple‐comparisons test (C, F, I, K, M, P, Q) and two‐way ANOVA with Tukey's multiple‐comparisons test (G, N). ***p < .001.

To elucidate the mechanism underlying the synergy between nitidine and Osi, we performed RNA sequencing on PC‐9 cells treated with Osi, nitidine, or a combination of both. Differential gene analysis was conducted to identify genes upregulated in Osi and nitidine compared to DMSO, and those further upregulated in the combination group. Similarly, genes downregulated in Osi and nitidine compared to DMSO were examined, with further downregulation observed in the combination treatment. Notably, we identified a persistent upregulation of NEBL, PLAT, GRIA2, FBXL17, PRKCA and CAMK1D, and a sustained downregulation of FUNDC1. These upregulated genes are not associated with cell death, while the downregulated FUNDC1 is involved in a non‐classical mitochondrial autophagy pathway (Figure 4A,B).

We therefore focused on FUNDC1, a receptor involved in non‐canonical mitophagy. RT‐qPCR confirmed that FUNDC1 mRNA was reduced following treatment with either Osi or nitidine and was further decreased by the combination (Figure 4C). Immunoblotting further confirmed that FUNDC1 protein levels were reduced in both PC‐9 and NCI‐H1975 cells (Figure 4D). By contrast, PINK1 remained largely unchanged, arguing against a dominant contribution from the PINK1/Parkin pathway under these conditions. To further define the mitophagy programme involved, we examined additional mitophagy‐related regulators. Neither BNIP3/BNIP3L (NIX) nor MARCH5 showed robust induction or consistent regulation under the same treatment conditions (Figure S6A–E), supporting the view that the observed response is not primarily mediated through these pathways.

We next assessed the effects of Osi and nitidine on mitophagy. Immunoblotting showed that p62 increased, whereas TOM20, TIM23 and LC3‐I/II decreased after treatment, with the combination showing the strongest effect (Figure 4D). mt‐Keima flow cytometry further showed that both Osi and nitidine reduced the proportion of cells with mitophagy events, and that the combination caused a further decrease (Figure 4E,F). The mtDNA/nDNA ratio showed the same pattern, with the combination again having the strongest effect (Figure 4G). Mitophagy dye staining also showed reduced signal intensity in both PC‐9 cells and EGFR‐mutant PDOs after either single treatment, with the greatest reduction in the combination group (Figure 4H–K).

Because inhibition of mitophagy is expected to perturb mitochondrial homeostasis, we next examined mitochondrial stress phenotypes. MitoSOX staining showed that both Osi and nitidine increased mtROS, whereas JC‐1 analysis showed a reduction in mitochondrial membrane potential; in both assays, the combination produced the strongest effect (Figure S6F–I). In addition, MitoTEMPO partially rescued the reduction in viability induced by Osi or nitidine (Figure S6J), supporting mtROS accumulation as a contributor to the cytotoxic response.

Finally, to determine whether FUNDC1 downregulation is functionally required for these phenotypes, we performed FUNDC1 overexpression rescue experiments. Restoring FUNDC1 partially recovered TOM20, TIM23 and LC3‐I/II expression, increased the proportion of cells with mitophagy events, normalised the mtDNA/nDNA ratio, restored mitophagy dye intensity, and attenuated the loss‐of‐viability phenotype induced by Osi or nitidine (Figure 4L–Q).

### 3.4 Osimertinib and nitidine converge on HIF‐1α suppression to downregulate FUNDC1‐dependent mitophagy

To investigate how FUNDC1 is downregulated upon Osi and nitidine treatment, we next examined the upstream regulatory mechanism. Given previous reports linking HIF‐1α/FUNDC1 signalling to mitophagy in other settings, we next asked whether this pathway is involved in the response observed here.^30^

**FIGURE 5:**
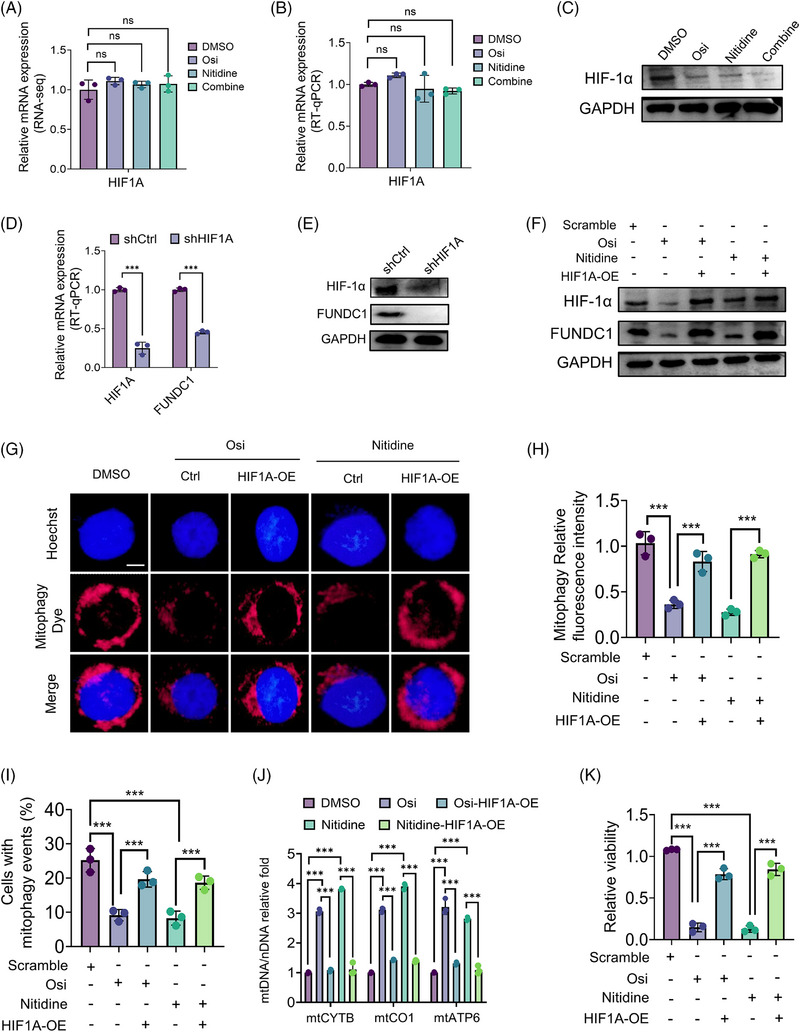
Osimertinib and nitidine converge on HIF‐1α suppression to downregulate FUNDC1‐dependent mitophagy. (A) Relative HIF1A mRNA levels derived from RNA‐seq analysis in PC‐9 cells treated with DMSO, Osi (100 nM), nitidine (10 µM), or the combination. (B) RT‐qPCR analysis of HIF1A mRNA expression in PC‐9 cells following the indicated treatments. (C) Immunoblot analysis of HIF‐1α protein levels in PC‐9 cells following the indicated treatments. (D) RT‐qPCR analysis of HIF1A and FUNDC1 mRNA expression in PC‐9 cells transduced with control or HIF1A‐targeting shRNA. (E) Immunoblot analysis of HIF‐1α and FUNDC1 protein levels in PC‐9 cells expressing control or HIF1A‐targeting shRNA. (F) Immunoblot analysis of HIF‐1α and FUNDC1 in PC‐9 cells expressing control vector or HIF1A overexpression (HIF1A‐OE) after treatment with Osi or nitidine. (G) Representative fluorescence images showing mitophagy dye signal in PC‐9 cells expressing control vector or HIF1A‐OE after treatment with Osi or nitidine; scale bar = 5 µm. (H) Quantification of the relative mitophagy fluorescence intensity in PC‐9 cells expressing control vector or HIF1A‐OE after treatment with Osi or nitidine. (I) Quantification of cells with mitophagy events in PC‐9‐mt‐Keima cells expressing control vector or HIF1A‐OE in PC‐9 cells expressing control vector or HIF1A‐OE after treatment with Osi or nitidine. (J) RT‐qPCR‐based quantification of the mtDNA/nDNA ratio in PC‐9 cells expressing control vector or HIF1A‐OE after treatment with Osi or nitidine. (K) Relative cell viability of control and HIF1A‐overexpressing PC‐9 cells after treatment with Osi or nitidine. Data are mean ± SEM. Statistical analyses were determined by one‐way ANOVA with Tukey's multiple‐comparisons test (A, B, H, I, K), unpaired two‐tailed Student's *t*‐test (D), and two‐way ANOVA with Tukey's multiple‐comparisons test (J). ns, not significant; ***p < .001.

RNA‐seq and RT‐qPCR did not show a significant decrease in HIF1A mRNA. Immunoblotting showed that both drugs reduced HIF‐1α protein levels, with the combination showing the strongest effect (Figure 5A–C). This result suggested that HIF‐1α may act upstream of FUNDC1. To test this, we knocked down HIF1A and found that loss of HIF1A reduced FUNDC1 expression at the RNA and protein levels (Figure 5D,E), suggesting that HIF‐1α helps maintain FUNDC1 expression in EGFR‐mutant NSCLC cells. We then examined whether restoring HIF‐1α could reverse the effects of Osi or nitidine. HIF1A overexpression partially restored FUNDC1 protein levels in cells treated with either drug (Figure 5F). It also restored mitophagy, as shown by the recovery of mitophagy dye intensity, the percentage of cells with mitophagy events, and the mtDNA/nDNA ratio (Figure 5G‐J). In addition, HIF1A overexpression partially rescued the reduction in cell viability caused by Osi or nitidine (Figure 5K). Together, these results show that reduced HIF‐1α protein is an important upstream event leading to FUNDC1 downregulation and inhibition of FUNDC1‐dependent mitophagy after Osi and nitidine treatment.

### 3.5 ABCB6 is required for nitidine‐induced suppression of HIF‐1α/FUNDC1‐mediated mitophagy, whereas BID contributes to downstream apoptotic response

**FIGURE 6:**
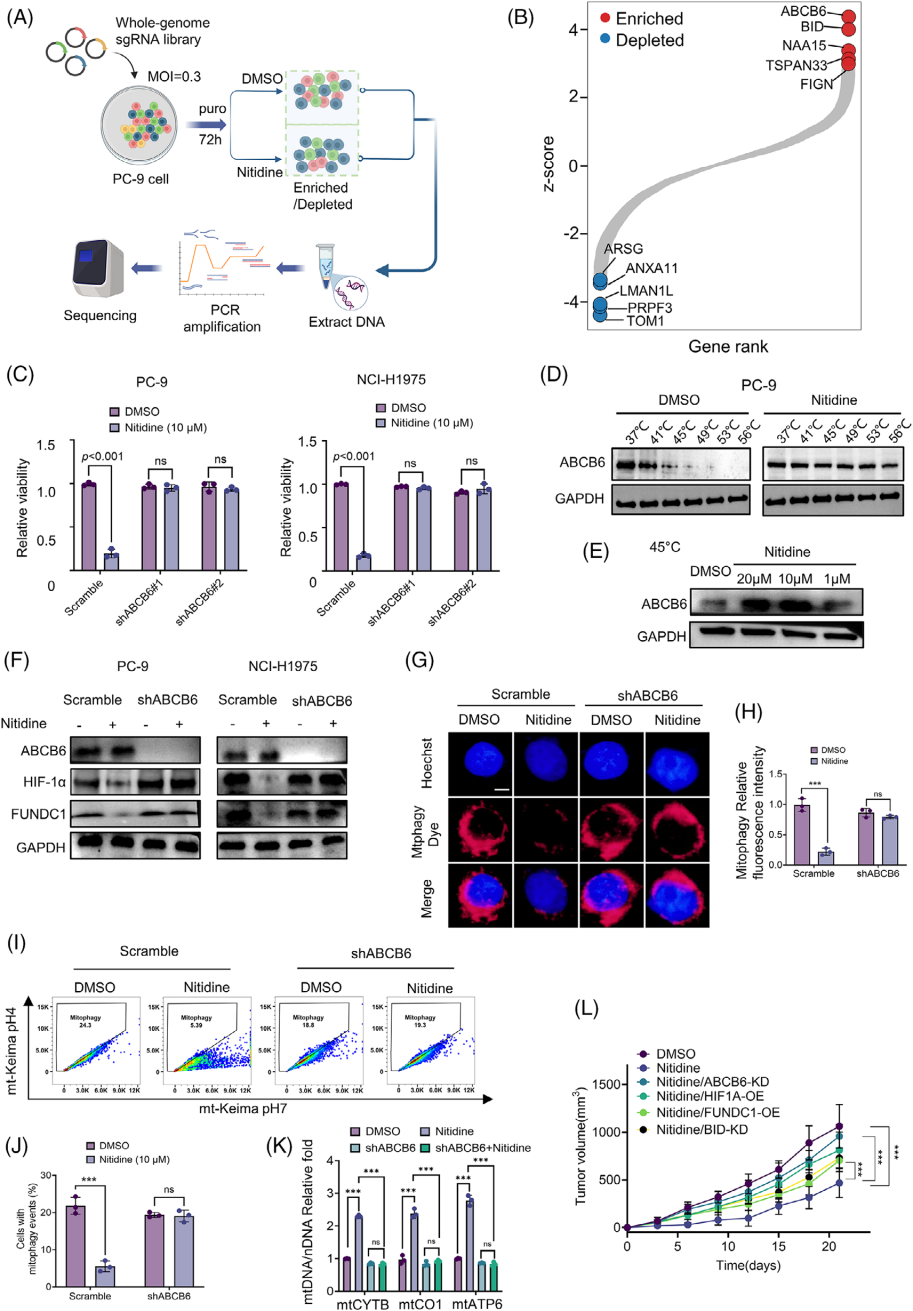
ABCB6 is required for nitidine‐induced suppression of HIF‐1α/FUNDC1‐mediated mitophagy. (A) Schematic of in vitro CRISPR screening. (B) Scatter plot showing gene‐level mean *Z*‐score changes in PC‐9 cells. Red indicates commonly enriched genes, and blue indicates depleted genes. (C) Relative cell viability of PC‐9 and NCI‐H1975 cells expressing either ABCB6 shRNA or control shRNA, followed by treatment with nitidine (10 µM). (D) Immunoblotting analysis of ABCB6 protein thermal stability by CETSA after treatment with DMSO or nitidine (10 µM) in PC‐9 cells. (E) Concentration‐dependent CETSA analysis of ABCB6 at 45°C in PC‐9 cells treated with the indicated concentrations of nitidine. (F) Immunoblot analysis of ABCB6, HIF‐1α and FUNDC1 in PC‐9 and NCI‐H1975 cells expressing control or ABCB6‐targeting shRNA and treated with DMSO or nitidine. (G) Representative fluorescence images showing mitophagy dye signal in control and ABCB6‐knockdown PC‐9 cells treated with DMSO or nitidine; scale bar = 5 µm. (H) Quantification of the relative mitophagy fluorescence intensity in control and ABCB6‐knockdown PC‐9 cells treated with DMSO or nitidine. (I) Representative mt‐Keima flow cytometry plots showing mitophagy in control and ABCB6‐knockdown PC‐9‐mt‐Keima cells treated with DMSO or nitidine. (J) Quantification of cells with mitophagy events in control and ABCB6‐knockdown PC‐9 cells treated with DMSO or nitidine. (K) RT‐qPCR‐based quantification of the mtDNA/nDNA ratio in control and ABCB6‐knockdown PC‐9 cells treated with DMSO or nitidine, measured using mtCYTB, mtCO1 and mtATP6. (L) Tumour growth curves from xenografts generated with control, ABCB6‐knockdown, HIF1A‐overexpression, FUNDC1‐overexpression or BID‐knockdown PC‐9 cells after nitidine treatment. Data are mean ± SEM from three independent experiments unless otherwise indicated. Statistical significance was determined by unpaired two‐tailed Student's *t*‐test (C, H, J), one‐way ANOVA with Tukey's multiple‐comparisons test (L), and two‐way ANOVA with Tukey's multiple‐comparisons test (K). ns, not significant; ***p < .001.

To identify mediators of the Nitidine response, we performed a genome‐wide CRISPR screen in PC‐9 cells exposed to nitidine and found ABCB6 and BID among the top enriched hits (Figure 6A,B). We first focused on ABCB6, as it has been implicated as a nitidine‐binding protein and was directly linked to the mitochondrial phenotype explored in this study.^31^ Functionally, ABCB6 knockdown significantly attenuated the growth‐inhibitory effect of nitidine in both PC‐9 and NCI‐H1975 cells (Figure 6C; Figure S7A), indicating that ABCB6 is important for the nitidine response.

We then examined target engagement using a CETSA. Nitidine increased the thermal stability of ABCB6 in both the temperature‐gradient and concentration‐gradient assays (Figure 6D,E), supporting direct interaction between nitidine and ABCB6 in EGFR‐mutant NSCLC cells.

Next, we asked whether ABCB6 is involved in the effect of nitidine on the HIF‐1α/FUNDC1 pathway. Without nitidine treatment, ABCB6 knockdown did not obviously change HIF‐1α or FUNDC1 levels; however, when ABCB6 knockdown cells were treated with nitidine, the drug‐induced suppression of HIF‐1α and FUNDC1 was markedly attenuated in both PC‐9 and NCI‐H1975 cells (Figure 6F). However, ABCB6 knockdown did not affect the ability of Osi to suppress HIF‐1α or FUNDC1 (Figure S7B). We further assessed whether ABCB6 knockdown affects nitidine‐induced mitophagy. ABCB6 knockdown clearly attenuated the inhibitory effect of nitidine on mitophagy, as shown by mitophagy dye staining, mt‐Keima flow cytometry and mtDNA/nDNA analysis (Figure 6G–K). These data indicate that nitidine‐induced suppression of HIF‐1α and FUNDC1 and HIF‐1α/FUNDC1‐mediated mitophagy depend on ABCB6.

The same screen also identified BID as an enriched hit, suggesting that it may be involved in the downstream cell‐death response to nitidine. BID knockdown partially attenuated nitidine‐induced loss of clonogenic growth and viability in both PC‐9 and NCI‐H1975 cells (Figure S7C,D). Moreover, annexin V/PI staining and cleaved caspase‐3 analysis further showed that nitidine‐induced apoptosis was attenuated when either ABCB6 or BID was knocked down (Figure S7E–G). In xenograft experiments, genetic perturbation of ABCB6, HIF1A, FUNDC1 or BID also weakened the tumour‐suppressive effect of nitidine, in agreement with the in vitro findings (Figure 6L; Figure S7H,I). These results support that nitidine suppresses the HIF‐1α/FUNDC1 pathway through ABCB6 and inhibits mitophagy, while BID‐associated apoptotic signalling contributes to the downstream cytotoxic response.

### 3.6 Osimertinib‐resistant NSCLC displays heightened sensitivity to nitidine

**FIGURE 7:**
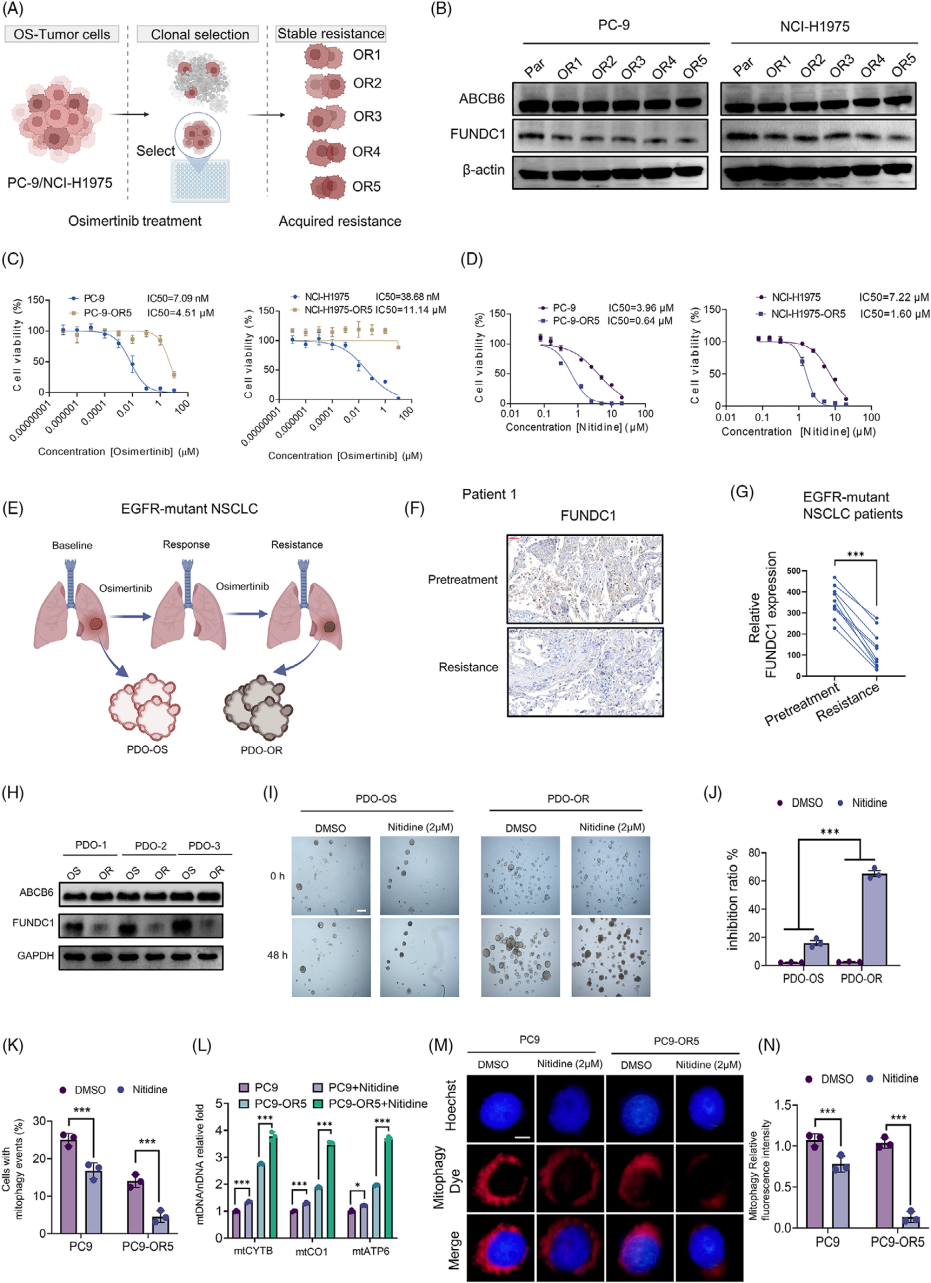
Osimertinib‐resistant NSCLC exhibits hypersensitivity to nitidine due to adaptive downregulation of FUNDC1. (A) Schematic of the establishment of Osi‐resistant cell lines, PC‐9‐OR and NCI‐H1975‐OR. (B) Immunoblotting of ABCB6 and FUNDC1 in Osi‐resistant cell lines PC‐9‐OR and NCI‐H1975‐OR, compared with their parental cell lines. (C) Cell viability of PC‐9, PC‐9‐OR, NCI‐H1975 and NCI‐H1975‐OR following treatment with the EGFR inhibitor Osi. (D) Cell viability of PC‐9, PC‐9‐OR, NCI‐H1975 and NCI‐H1975‐OR following treatment with nitidine. (E) Schematic of the establishment of Osi‐resistant EGFR‐mutant patient‐derived organoids (PDOs). (F) Representative immunohistochemical images of FUNDC1 in paired patient tumour samples collected before treatment and after the development of Osi resistance; scale bars = 25 µm. (G) Quantification of FUNDC1 immunohistochemical signal in paired clinical samples (*n* = 10). (H) Immunoblot analysis of ABCB6 and FUNDC1 in paired PDO samples before treatment (OS) and after Osi resistance (OR) (*n* = 3). (I) Representative bright‐field images of paired PDO‐OS and PDO‐OR cultures treated with DMSO or nitidine (2 µM) for the indicated times; scale bars = 200 µm. (J) Quantification of the growth‐inhibitory effect of nitidine in paired PDO‐OS and PDO‐OR models. (K) Quantification of cells with mitophagy events in parental PC‐9 and PC‐9‐OR5 cells treated with DMSO or nitidine. (L) RT‐qPCR‐based quantification of the mtDNA/nDNA ratio in parental PC‐9 and PC‐9‐OR5 cells treated with DMSO or nitidine, measured using mtCYTB, mtCO1 and mtATP6. (M) Representative fluorescence images showing mitophagy dye signal in parental PC‐9 and PC‐9‐OR5 cells treated with DMSO or nitidine (2 µM); scale bars = 5 µm. (N) Quantification of the relative mitophagy fluorescence intensity parental PC‐9 and PC‐9‐OR5 cells treated with DMSO or nitidine (2 µM). Data are mean ± SEM from three independent experiments unless otherwise indicated. Statistical significance was determined by paired two‐tailed Student's *t*‐test (G), unpaired two‐tailed Student's *t*‐test (J, K, N), and two‐way ANOVA with Tukey's multiple‐comparisons test (L). ns, not significant; ***p < .001.

To assess whether the Osi‐induced downregulation of FUNDC1 is maintained upon acquisition of resistance, we generated Osi‐resistant subclones from EGFR‐mutant PC‐9 and NCI‐H1975 NSCLC cells (Figure 7A). Immunoblotting revealed a marked reduction in FUNDC1 protein expression in the resistant subclones compared to their parental cells, while ABCB6 protein levels remained unchanged (Figure 7B). Notably, nitidine exhibited a stronger growth‐inhibitory effect on Osi‐resistant PC‐9‐OR and NCI‐H1975‐OR cells than on their parental counterparts (Figure 7C,D). We then established paired EGFR‐mutant NSCLC patient‐derived organoids (PDOs) from the same patient—before Osi treatment (PDO‐OS) and after the development of Osi resistance (PDO‐OR) (Figure 7E). In paired tumour tissues, FUNDC1 immunohistochemistry showed a consistent reduction in FUNDC1 expression in the resistant state (Figure 7F,G; Figure S8A). Owing to the limited number of paired resistant specimens currently available, we did not identify an obvious association between FUNDC1 expression and specific resistance mechanisms in this cohort. Similarly, immunoblot analysis of paired PDO samples showed lower FUNDC1 expression after resistance, whereas ABCB6 remained relatively stable (Figure 7H). Functionally, nitidine exerted a stronger growth‐inhibitory effect in PDO‐OR than in the matched PDO‐OS models (Figure 7I,J).

We next asked whether the increased nitidine sensitivity of resistant cells was accompanied by further suppression of mitophagy. Compared with parental PC‐9 cells, PC‐9‐OR5 cells exhibited a greater reduction in the percentage of cells with mitophagy events and in mitophagy dye intensity following nitidine treatment, together with a higher mtDNA/nDNA ratio, consistent with impaired mitochondrial clearance (Figure 7K–N).

Moreover, Osi treatment reduced FUNDC1 expression in PC‐9 and NCI‐H1975 cells within 48 h, and this reduction persisted in drug‐tolerant persister cells (DTPCs), while ABCB6 levels remained unaffected (Figure S8B). Collectively, these data demonstrate that the early and sustained downregulation of FUNDC1 during Osi treatment, maintained in drug‐tolerant and fully resistant states, creates a collateral vulnerability that renders resistant cells more sensitive to nitidine monotherapy.

## 4 DISCUSSION

The persistent challenge of acquired resistance to Osi in EGFR‐mutant NSCLC has driven the development of various combination strategies aimed at extending therapeutic efficacy.^3^, ^32^, ^33^, ^34^, ^35^ Our study identified a synergistic regimen by combining the traditional medicine XHP with Osi in EGFR‐mutant NSCLC. We established nitidine as the active component of XHP, which synergises with Osi by modulating the HIF‐1α/FUNDC1‐mediated mitophagy, thereby enhancing mitochondrial stress and cell death.

Resistance mechanisms to EGFR‐TKIs are notably heterogeneous, involving on‐target secondary mutations, off‐target bypass activation (such as MET or HER2 amplification) and histologic transformation.^36^ While these pathways have been extensively studied, the role of mitochondrial homeostasis—specifically mitophagy,^37^, ^38^ which is responsible for removing damaged mitochondria—has remained largely unexplored, representing a critical knowledge gap.^39^, ^40^, ^41^, ^42^, ^43^, ^44^ Although FUNDC1 has been shown to contribute to drug resistance in therapies like sorafenib and carboplatin,^45^, ^46^ its role in modulating the response to EGFR‐targeted therapies has not been well established. In our study, we demonstrated that both the natural compound nitidine and Osi suppress FUNDC1‐mediated mitophagy. This mechanistic convergence identifies the FUNDC1‐mediated mitophagy pathway as a druggable vulnerability.^47^ More importantly, we observed adaptive downregulation of FUNDC1 in Osi‐resistant models. Crucially, the reduced baseline expression of FUNDC1 renders resistant cells highly dependent on its residual function, making them hypersensitive to complete blockade by nitidine. This finding highlights FUNDC1 as an indispensable orchestrator of a targetable vulnerability in Osi‐resistant EGFR‐mutant NSCLC.

An important result of this work is that HIF‐1α sits upstream of FUNDC1 in this setting. We found that Osi and nitidine reduced HIF‐1α protein, but not HIF1A mRNA. Restoring HIF‐1α partially restored FUNDC1 expression, mitophagy and cell viability. These data support that a decrease in HIF‐1α protein contributes to FUNDC1 downregulation, which in turn weakens mitophagy. This gives a clearer explanation for why the two drugs act together, because they meet at the level of HIF‐1α/FUNDC1‐mediated mitophagy rather than only at the level of general cytotoxic stress.

While ABCB6 is known for its role in porphyrin transport, recent studies have implicated it in modulating cell death in multiple myeloma and colorectal cancer.^31^, ^37^

Our data also make the role of ABCB6 clearer. The results do not support ABCB6 as a general regulator of basal HIF‐1α/FUNDC1 expression. Instead, ABCB6 is specifically required for the nitidine response. When nitidine is absent, ABCB6 knockdown does not obviously change HIF‐1α or FUNDC1. When nitidine is present, however, the suppression of HIF‐1α, FUNDC1 and mitophagy becomes much weaker in ABCB6‐knockdown cells. Together with the CETSA results, this shows that ABCB6 defines the nitidine‐specific branch of the pathway. This is an important point, because it means that nitidine is not simply causing a non‐specific mitochondrial effect.

Notably, FUNDC1 downregulation is maintained in the resistant state. In resistant cell lines, paired patient tissues and paired PDOs, FUNDC1 expression remained lower after the development of Osi resistance, whereas ABCB6 remained relatively stable. At the same time, resistant cells and PDOs showed increased sensitivity to nitidine. We do not interpret these data to mean that reduced FUNDC1 alone drives resistance. Instead, they suggest that chronic EGFR inhibition is associated with an adaptive low‐FUNDC1 state in which cells remain dependent on residual mitochondrial quality control. In this setting, further suppression of mitophagy by nitidine may push mitochondrial stress beyond a tolerable threshold and render resistant cells more vulnerable to cell death.^48^, ^49^

From a translational point of view, these findings suggest two possible uses of nitidine. One is a combination therapy with Osi, with the aim of deepening suppression of a mitophagy‐dependent survival pathway and extending treatment benefit. The other is post‐resistance treatment, where nitidine may be useful in tumours that have entered an adaptive FUNDC1‐low state. The second possibility may be particularly interesting, because treatment after Osi progression is increasingly shaped by resistance context and by the search for mechanism‐based vulnerabilities.

Our genome‐wide CRISPR screen and follow‐up experiments suggest that BID‐associated apoptotic signalling contributes to the nitidine response. However, we did not test whether Osi‐induced apoptosis shows the same dependence on BID, nor did we examine how BID relates to established EGFR‐TKI‐associated apoptotic mediators such as BIM or PUMA in this model. We do not interpret these findings as evidence for a single shared apoptotic pathway. Instead, our data support a role for BID‐associated apoptosis in the nitidine response, whereas the broader relationship between this signal and EGFR‐TKI‐induced apoptosis remains unclear. By comparison, suppression of HIF‐1α/FUNDC1‐dependent mitophagy was the clearest point of convergence in our study.

This study also has several limitations. First, the precise biochemical link between ABCB6 and HIF‐1α/FUNDC1 regulation is still not defined. Second, the paired clinical cohort and PDO set remain limited in size, which restricts survival analysis and subgroup analysis across different resistance mechanisms. Third, although our selectivity and tolerability data support nitidine in the current preclinical setting, a more quantitative pharmacological framework linking dose, exposure and efficacy remains to be established.

In summary, our study shows that Osi and nitidine converge on HIF‐1α suppression to downregulate FUNDC1‐dependent mitophagy in EGFR‐mutant NSCLC. We further identify ABCB6 as a required mediator of the nitidine‐induced HIF‐1α/FUNDC1 response and show that adaptive FUNDC1 downregulation in resistant models creates increased sensitivity to nitidine monotherapy. These findings support mitophagy‐targeted intervention as a potential strategy both to enhance upfront EGFR‐TKI efficacy and to exploit a resistance‐associated vulnerability after Osi treatment.

## AUTHOR CONTRIBUTIONS

**Fan Xu, Xiaoshan Wang, Min Li, Fanming Kong, Qihong Huang, Xin Cao and Ying Xue** conceived and designed the study. **Fan Xu, Xiaoshan Wang and Min Li** performed experiments and acquired data. **Yi Li, Xiaojuan Li and Qingqing Yan** analysed and interpreted the data. **Fan Xu and Xiaoshan Wang** drafted the manuscript. All authors critically revised the manuscript, approved the final version, and agree to be accountable for all aspects of the work.

## CONFLICT OF INTEREST STATEMENT

The authors declare no conflicts of interest.

## ETHICS STATEMENT

All patients included in the study have signed informed consent in compliance with the Declaration of Helsinki. This study was approved by the First Medical University Research Ethics Committee of the Shandong Provincial Hospital Affiliated to Shandong First Medical University, Shandong.

## Supporting information



Supporting Information

Supporting Information

## Data Availability

The data supporting the findings of this study are available within the article and its Supporting Information files. Additional data are available from the corresponding authors upon reasonable request.
