# Supplementary material for: Convergent targeting of FUNDC1‐dependent mitophagy sensitises and overcomes resistance to EGFR inhibition
Source: Clin Transl Med. 2026 May 18;16(5):e70685. doi: 10.1002/ctm2.70685 (PMC13184547; doi:10.1002/ctm2.70685)
Supplement: Supplementary file 1 — Supporting Information [file CTM2-16-e70685-s002.docx]

**Figure S1.** Forest plots of meta-analyses evaluating the efficacy and safety of Xihuang Pill combined with conventional therapy versus control.

A-B. Complete response (CR): **(A)** overall effect; **(B)** subgroup by cancer type. C-D. Partial response (PR): **(C)** overall effect; **(D)** subgroup by cancer type. **E-F.** Objective response rate (ORR): **(E)** overall effect; **(F)** subgroup by cancer type.

**Figure S2. Additional validation of the antitumor activity of Xihuang Pill and its combination with Osimertinib in EGFR-mutant NSCLC models.**

**A.** Inhibition efficacy of EGFR-mutant NSCLC patient-derived organoids (PDOs) following treatment with varying concentrations of Xihuang Pill (XHP) (5 %, 10 %, 20 %). **B.** Clonogenic assay of PC-9 and NCI-H1975 cells following treatment with varying concentrations of XHP (5 %, 10 %, 20 %). **C.** Clonogenic assay of PC-9 and NCI-H1975 cells following treatment with Osi, XHP-10% or the combined regimen. **D.** Inhibition efficacy of EGFR-mutant NSCLC PDOs following treatment with Osi, XHP or the combined regimen.

Data are presented as mean ± s.e.m. Statistical significance was determined by one-way ANOVA with Tukey’s multiple-comparisons test (**A, B, D**). *P < 0.05, **P < 0.01, ***P < 0.001.

**Figure S3. Nitidine does not show clear synergy with Hypericin or Camptothecin.**
**A.** ZIP synergy analysis of Nitidine in combination with Hypericin in NCI-H1975 cells. Left, dose–response matrix showing inhibition efficacy across the indicated concentration combinations. Right, three-dimensional ZIP synergy distribution plot. **B.** ZIP synergy analysis of Nitidine in combination with Camptothecin in NCI-H1975 cells. Left, dose–response matrix showing inhibition efficacy across the indicated concentration combinations. Right, three-dimensional ZIP synergy distribution plot. ZIP scores < 10 were interpreted as indicating the absence of clear synergy.

**Figure S4. Nitidine shows a measurable in vitro selectivity window and is well tolerated in vivo**.

**A.** Dose–response curves showing the viability of normal human cell lines (THLE2, HBE and MRC-5) following treatment with the indicated concentrations of Nitidine. **B.** Summary table showing the IC50 values of Nitidine in EGFR-mutant NSCLC cell lines and normal human cell lines, together with the estimated therapeutic index calculated relative to PC-9 or NCI-H1975 cells. **C.** Serum biochemical analyses of ALT, AST, CREA, and CK-MB in mice treated with DMSO or Nitidine. **D.** Representative hematoxylin and eosin (H&E) staining of major organs, including heart, liver, spleen, lung and kidney, from mice treated with DMSO or Nitidine, Scale bars = 200 μm.

Data are mean ± s.e.m. from three independent experiments unless otherwise indicated. Statistical significance was determined by unpaired two-tailed Student’s t-test (**C**). ns, not significant.

**Figure S5.** **Additional validation of the sensitivity of EGFR-mutant NSCLC PDOs to Nitidine and Osimertinib.**

**A.** Patient-derived EGFR mutant NSCLC organoids following Osimertinib or Nitidine, or combined treatment, scale bar = 200 μm. **B.** Inhibition efficacy of EGFR-mutant NSCLC patient-derived organoids (PDOs) following treatment with Nitidine. **C.** Inhibition efficacy of EGFR-mutant NSCLC PDOs following treatment with Osimertinib.

**Figure S6. BNIP3/BNIP3L and MARCH5 are not markedly altered, whereas mitochondrial stress contributes to the cytotoxic effect of Osimertinib plus Nitidine.**

**A.** Relative BNIP3 and BNIP3L mRNA levels derived from RNA-seq analysis in PC-9 cells following treatment with DMSO, Osimertinib (Osi), Nitidine, or the combination. **B.** RT–qPCR analysis of BNIP3 and BNIP3L mRNA expression in PC-9 cells following treatment with DMSO, Osi, Nitidine, or the combination. **C.** Immunoblot analysis of BNIP3 and BNIP3L protein levels in PC-9 cells following treatment with DMSO, Osi, Nitidine, or the combination. **D.** RNA-seq analysis of MARCHF5 mRNA expression in PC-9 cells following treatment with DMSO, Osi, Nitidine, or the combination. **E.** Immunoblot analysis of MARCH5 protein levels in PC-9 cells following treatment with DMSO, Osi, Nitidine, or the combination. **F.** Representative fluorescence images showing MitoSOX signal in PC-9 cells following treatment with Osi, Nitidine, or the combination, scale bars = 25 μm. **G.** Quantification of the relative MitoSOX fluorescence following treatment with DMSO, Osi, Nitidine, or the combination. **H.** Representative JC-1 fluorescence images showing monomeric (green) and aggregated (red) JC-1 signal in PC-9 cells after treatments, scale bars = 25 μm. **I.** Quantification of the JC-1 red/green fluorescence ratio. **J**. Relative cell viability of PC-9 cells under the indicated treatment conditions with or without MitoTEMPO.

Data are mean ± s.e.m. from three independent experiments unless otherwise indicated. Statistical significance was determined by one-way ANOVA with Tukey’s multiple-comparisons test (**A, B, D, G, I, J**). ns, not significant; **P < 0.01, ***P < 0.001.

**Figure S7. ABCB6 and BID contribute to Nitidine-induced growth suppression and apoptotic signaling.**

**A.** Representative colony-formation assays of PC-9 and NCI-H1975 cells expressing control or ABCB6-targeting shRNAs following treatment with DMSO or Nitidine. **B.** Immunoblot analysis of ABCB6, HIF-1α and FUNDC1 in PC-9 cells expressing control or ABCB6-targeting shRNA and treated with DMSO or Osimertinib. **C.** Representative colony-formation assays of PC-9 and NCI-H1975 cells expressing control or BID-targeting shRNAs following treatment with DMSO or Nitidine. **D.** Relative cell viability of PC-9 and NCI-H1975 cells expressing control or BID-targeting shRNAs following treatment with Nitidine (10 μM). **E.** Immunoblot analysis of total caspase-3 and cleaved caspase-3 in PC-9 and NCI-H1975 cells expressing control, ABCB6-targeting, or BID-targeting shRNAs following treatment with Nitidine. **F.** Representative Annexin V/PI flow-cytometry plots of PC-9 cells under the indicated genetic perturbation and treatment conditions. **G.** Quantification of the apoptotic fraction. **H.** Immunoblot analysis of ABCB6, HIF-1α and FUNDC1 in xenograft tumor tissues from the indicated treatment groups. **I.** Immunoblot analysis of ABCB6, HIF-1α, and FUNDC1 in tumor tissues from xenografts the indicated treatment groups.

Data are mean ± s.e.m. from three independent experiments unless otherwise indicated. Statistical significance was determined by one-way ANOVA with Tukey’s multiple-comparisons test (**D, G**). ns, not significant; ***P < 0.001.

**Figure S8. Additional validation of adaptive FUNDC1 downregulation in Osimertinib-resistant models.**

**A.** Clinical metadata table for paired patient samples included in this study, showing sex, age, disease status at recurrence or progression, EGFR genotype and/or acquired alteration, and available co-alterations. **B.** Immunoblotting of ABCB6 and FUNDC1 in PC-9 and NCI-H1975 cells following Osi treatment at the indicated time points, as well as in drug-tolerant persister cells (DTPCs).
