# Supplementary material for: Convergent targeting of FUNDC1‐dependent mitophagy sensitises and overcomes resistance to EGFR inhibition
Source: Clin Transl Med. 2026 May 18;16(5):e70685. doi: 10.1002/ctm2.70685 (PMC13184547; doi:10.1002/ctm2.70685)
Supplement: Supplementary file 2 — Supporting Information [file CTM2-16-e70685-s001.pdf]

Figure S1

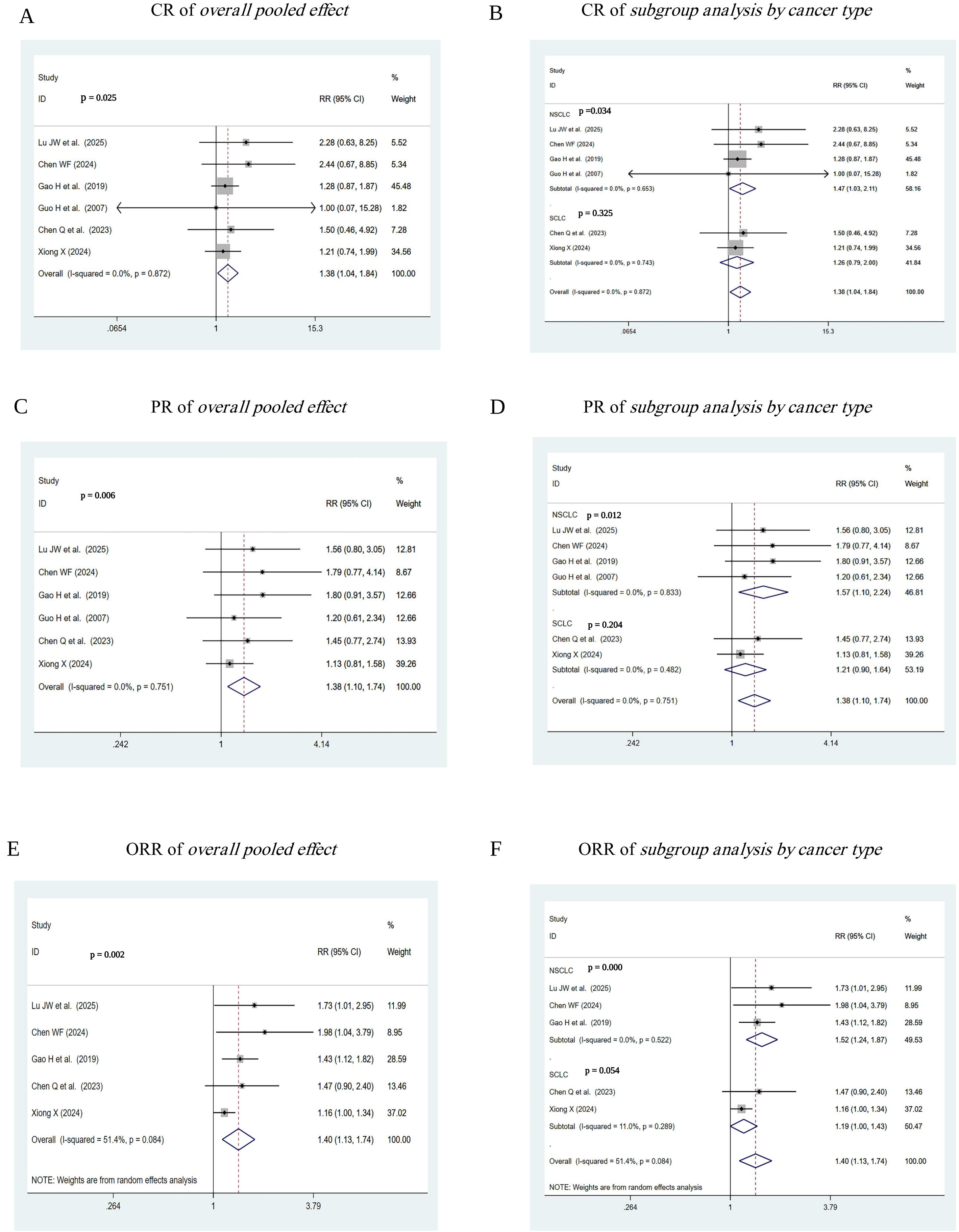

A
CR of *overall pooled effect*
Study
ID
p = 0.025
RR (95% CI)
%
Weight
Lu JW et al. (2025) 2.28 (0.63, 8.25) 5.52
Chen WF (2024) 2.44 (0.67, 8.85) 5.34
Gao H et al. (2019) 1.28 (0.87, 1.87) 45.48
Guo H et al. (2007) 1.00 (0.07, 15.28) 1.82
Chen Q et al. (2023) 1.50 (0.46, 4.92) 7.28
Xiong X (2024) 1.21 (0.74, 1.99) 34.56
Overall (I-squared = 0.0%, p = 0.872) 1.38 (1.04, 1.84) 100.00
.0654
1
15.3
B
CR of *subgroup analysis by cancer type*
Study
ID
RR (95% CI)
%
Weight
NSCLC p =0.034
Lu JW et al. (2025) 2.28 (0.63, 8.25) 5.52
Chen WF (2024) 2.44 (0.67, 8.85) 5.34
Gao H et al. (2019) 1.28 (0.87, 1.87) 45.48
Guo H et al. (2007) 1.00 (0.07, 15.28) 1.82
Subtotal (I-squared = 0.0%, p = 0.653) 1.47 (1.03, 2.11) 58.16
SCLC p = 0.325
Chen Q et al. (2023) 1.50 (0.46, 4.92) 7.28
Xiong X (2024) 1.21 (0.74, 1.99) 34.56
Subtotal (I-squared = 0.0%, p = 0.743) 1.26 (0.79, 2.00) 41.84
Overall (I-squared = 0.0%, p = 0.872) 1.38 (1.04, 1.84) 100.00
.0654
1
15.3
C
PR of *overall pooled effect*
Study
ID
p = 0.006
RR (95% CI)
%
Weight
Lu JW et al. (2025) 1.56 (0.80, 3.05) 12.81
Chen WF (2024) 1.79 (0.77, 4.14) 8.67
Gao H et al. (2019) 1.80 (0.91, 3.57) 12.66
Guo H et al. (2007) 1.20 (0.61, 2.34) 12.66
Chen Q et al. (2023) 1.45 (0.77, 2.74) 13.93
Xiong X (2024) 1.13 (0.81, 1.58) 39.26
Overall (I-squared = 0.0%, p = 0.751) 1.38 (1.10, 1.74) 100.00
.242
1
4.14
D
PR of *subgroup analysis by cancer type*
Study
ID
RR (95% CI)
%
Weight
NSCLC p = 0.012
Lu JW et al. (2025) 1.56 (0.80, 3.05) 12.81
Chen WF (2024) 1.79 (0.77, 4.14) 8.67
Gao H et al. (2019) 1.80 (0.91, 3.57) 12.66
Guo H et al. (2007) 1.20 (0.61, 2.34) 12.66
Subtotal (I-squared = 0.0%, p = 0.833) 1.57 (1.10, 2.24) 46.81
SCLC p = 0.204
Chen Q et al. (2023) 1.45 (0.77, 2.74) 13.93
Xiong X (2024) 1.13 (0.81, 1.58) 39.26
Subtotal (I-squared = 0.0%, p = 0.482) 1.21 (0.90, 1.64) 53.19
Overall (I-squared = 0.0%, p = 0.751) 1.38 (1.10, 1.74) 100.00
.242
1
4.14
E
ORR of *overall pooled effect*
Study
ID
p = 0.002
RR (95% CI)
%
Weight
Lu JW et al. (2025) 1.73 (1.01, 2.95) 11.99
Chen WF (2024) 1.98 (1.04, 3.79) 8.95
Gao H et al. (2019) 1.43 (1.12, 1.82) 28.59
Chen Q et al. (2023) 1.47 (0.90, 2.40) 13.46
Xiong X (2024) 1.16 (1.00, 1.34) 37.02
Overall (I-squared = 51.4%, p = 0.084) 1.40 (1.13, 1.74) 100.00
NOTE: Weights are from random effects analysis
.264
1
3.79
F
ORR of *subgroup analysis by cancer type*
Study
ID
RR (95% CI)
%
Weight
NSCLC p = 0.000
Lu JW et al. (2025) 1.73 (1.01, 2.95) 11.99
Chen WF (2024) 1.98 (1.04, 3.79) 8.95
Gao H et al. (2019) 1.43 (1.12, 1.82) 28.59
Subtotal (I-squared = 0.0%, p = 0.522) 1.52 (1.24, 1.87) 49.53
SCLC p = 0.054
Chen Q et al. (2023) 1.47 (0.90, 2.40) 13.46
Xiong X (2024) 1.16 (1.00, 1.34) 37.02
Subtotal (I-squared = 11.0%, p = 0.289) 1.19 (1.00, 1.43) 50.47
Overall (I-squared = 51.4%, p = 0.084) 1.40 (1.13, 1.74) 100.00
NOTE: Weights are from random effects analysis
.264
1
3.79

Figure S2

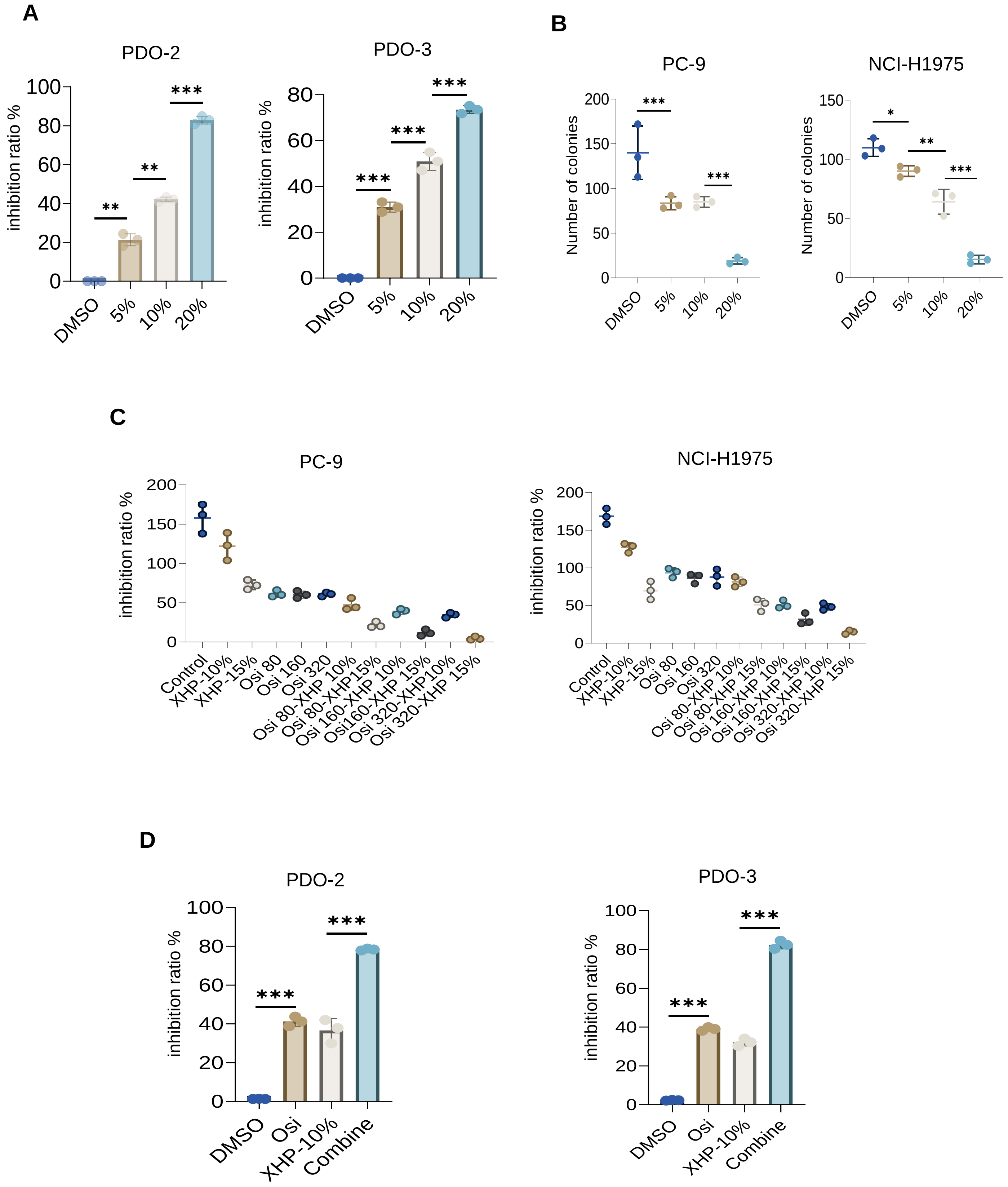

A
PDO-2
inhibition ratio %
100
80
60
40
20
0
DMSO
5%
10%
20%
**
**
***
PDO-3
inhibition ratio %
80
60
40
20
0
DMSO
5%
10%
20%
***
***
***
B
PC-9
Number of colonies
200
150
100
50
0
DMSO
5%
10%
20%
***
***
NCI-H1975
Number of colonies
150
100
50
0
DMSO
5%
10%
20%
*
**
***
C
PC-9
inhibition ratio %
200
150
100
50
0
Control
XHP-10%
XHP-15%
Osi 80
Osi 160
Osi 320
Osi 80-XHP 10%
Osi 80-XHP15%
Osi 160-XHP 10%
Osi160-XHP 15%
Osi 320-XHP10%
Osi 320-XHP 15%
NCI-H1975
inhibition ratio %
200
150
100
50
0
Control
XHP-10%
XHP-15%
Osi 80
Osi 160
Osi 320
Osi 80-XHP 10%
Osi 80-XHP 15%
Osi 160-XHP 10%
Osi 160-XHP 15%
Osi 320-XHP 10%
Osi 320-XHP 15%
D
PDO-2
inhibition ratio %
100
80
60
40
20
0
DMSO
Osi
XHP-10%
Combine
***
***
PDO-3
inhibition ratio %
100
80
60
40
20
0
DMSO
Osi
XHP-10%
Combine
***
***

Figure S3

A

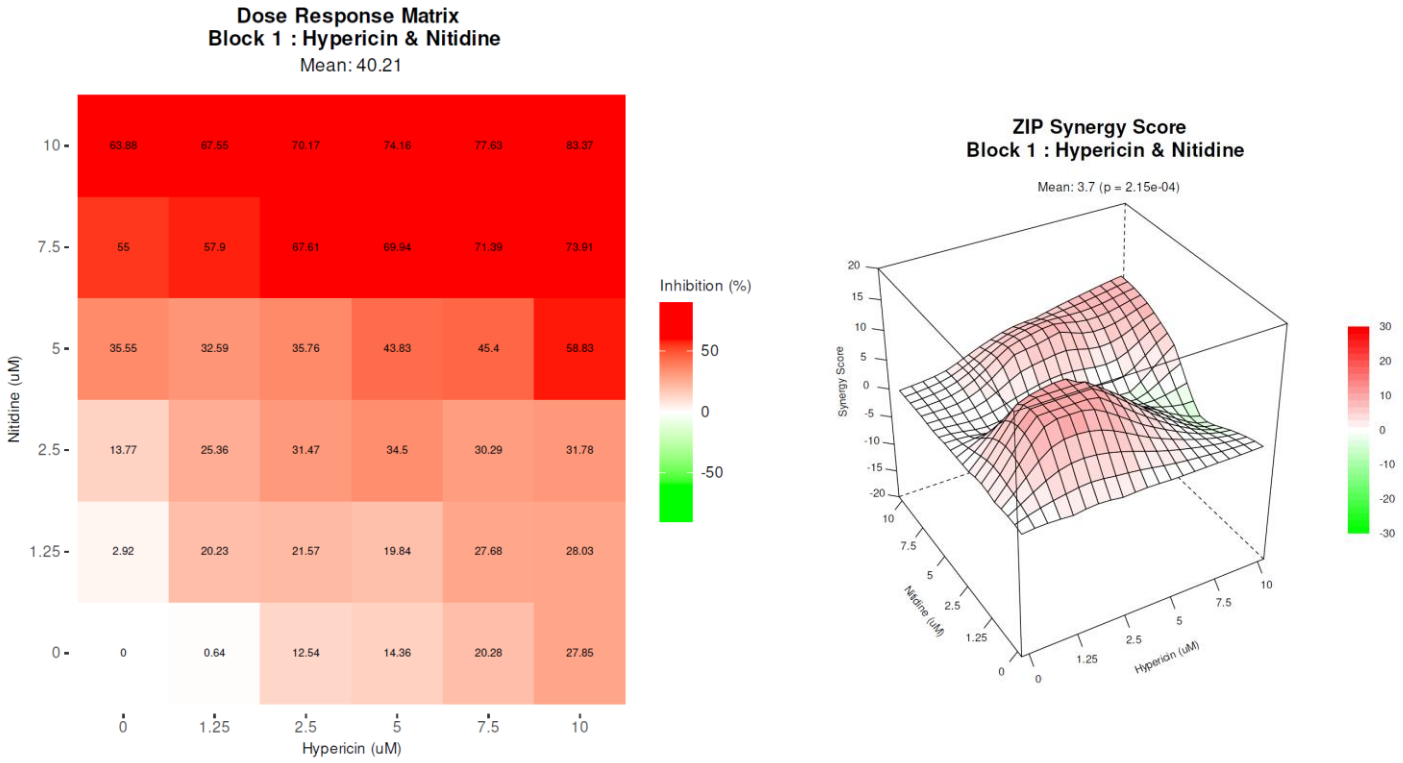

Dose Response Matrix
Block 1 : Hypericin & Nitidine
Mean: 40.21
63.88 67.55 70.17 74.16 77.63 83.37
55 57.9 67.61 69.94 71.39 73.91
35.55 32.59 35.76 43.83 45.4 58.83
13.77 25.36 31.47 34.5 30.29 31.78
2.92 20.23 21.57 19.84 27.68 28.03
0 0.64 12.54 14.36 20.28 27.85
Nitidine (uM)
Hypericin (uM)
Inhibition (%)
ZIP Synergy Score
Block 1 : Hypericin & Nitidine
Mean: 3.7 (p = 2.15e-04)
Synergy Score
Nitidine (uM)
Hypericin (uM)

B

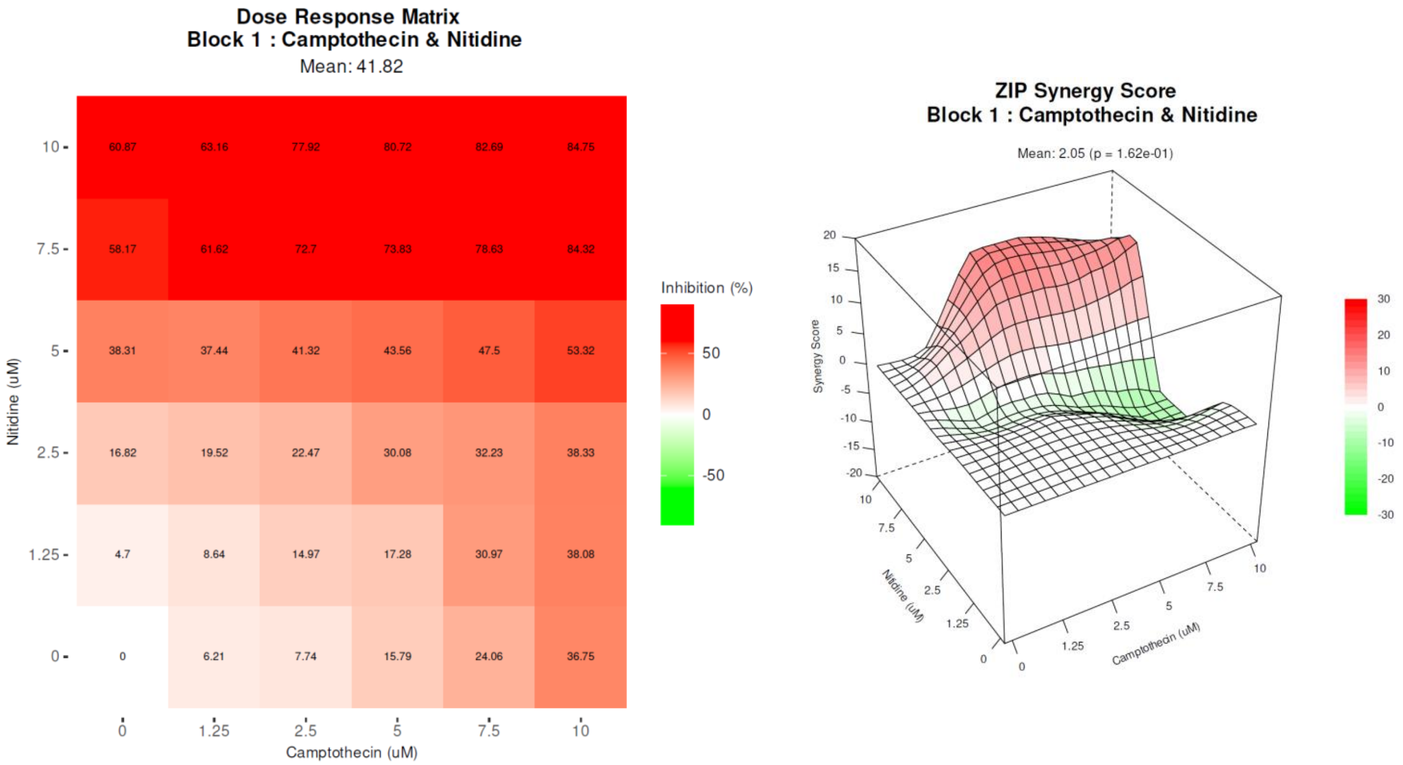

Dose Response Matrix
Block 1 : Camptothecin & Nitidine
Mean: 41.82
60.87 63.16 77.92 80.72 82.69 84.75
58.17 61.62 72.7 73.83 78.63 84.32
38.31 37.44 41.32 43.56 47.5 53.32
16.82 19.52 22.47 30.08 32.23 38.33
4.7 8.64 14.97 17.28 30.97 38.08
0 6.21 7.74 15.79 24.06 36.75
Nitidine (uM)
Camptothecin (uM)
Inhibition (%)
ZIP Synergy Score
Block 1 : Camptothecin & Nitidine
Mean: 2.05 (p = 1.62e-01)
Synergy Score
Nitidine (uM)
Camptothecin (uM)

Figure S4

A

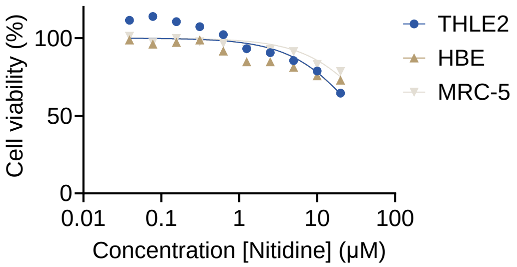

B

Cell line	IC50 (μM)	Therapeutic index (to PC-9)	Therapeutic index (to NCI-H1975)
PC-9	5.252	--	--
NCI-H1975	6.995	--	--
THLE2	35.45	6.750	5.068
HBE	33.27	6.335	4.756
MRC-5	59.98	11.420	8.575

C

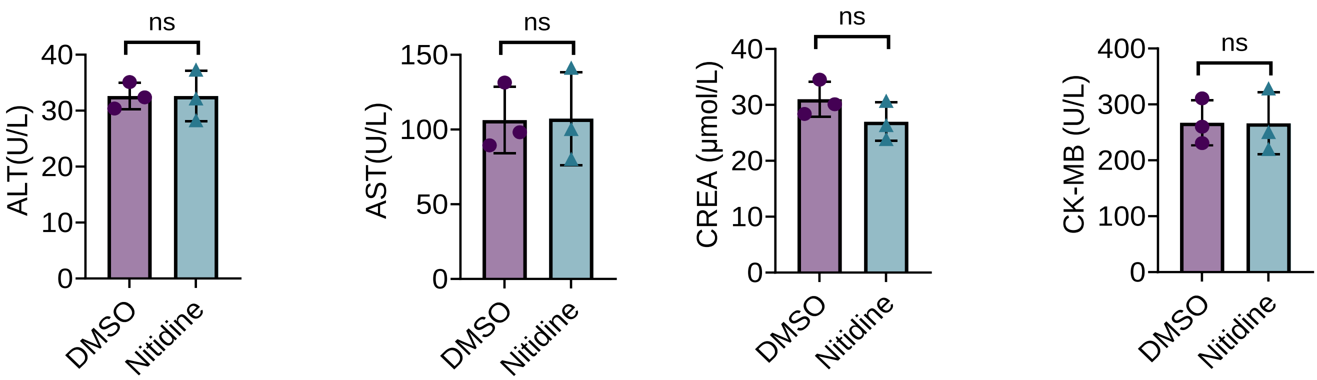

D

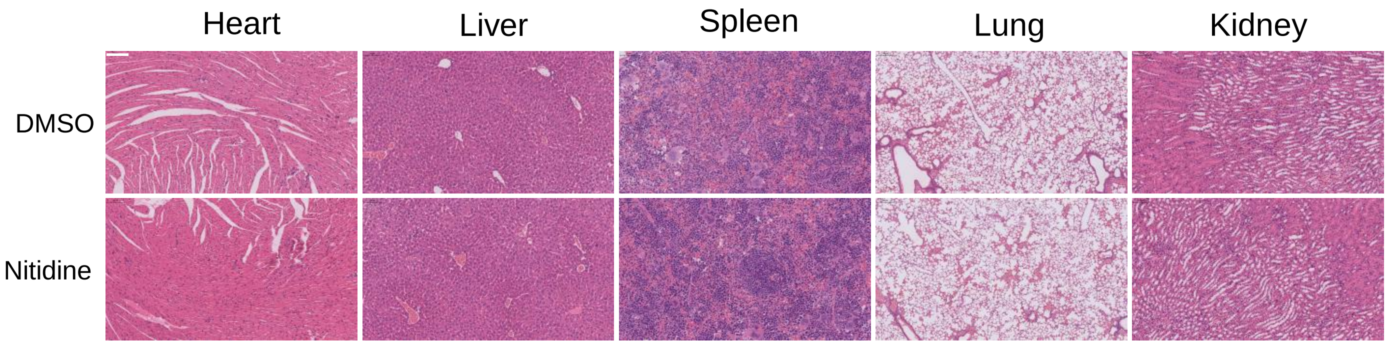

Figure S5

A

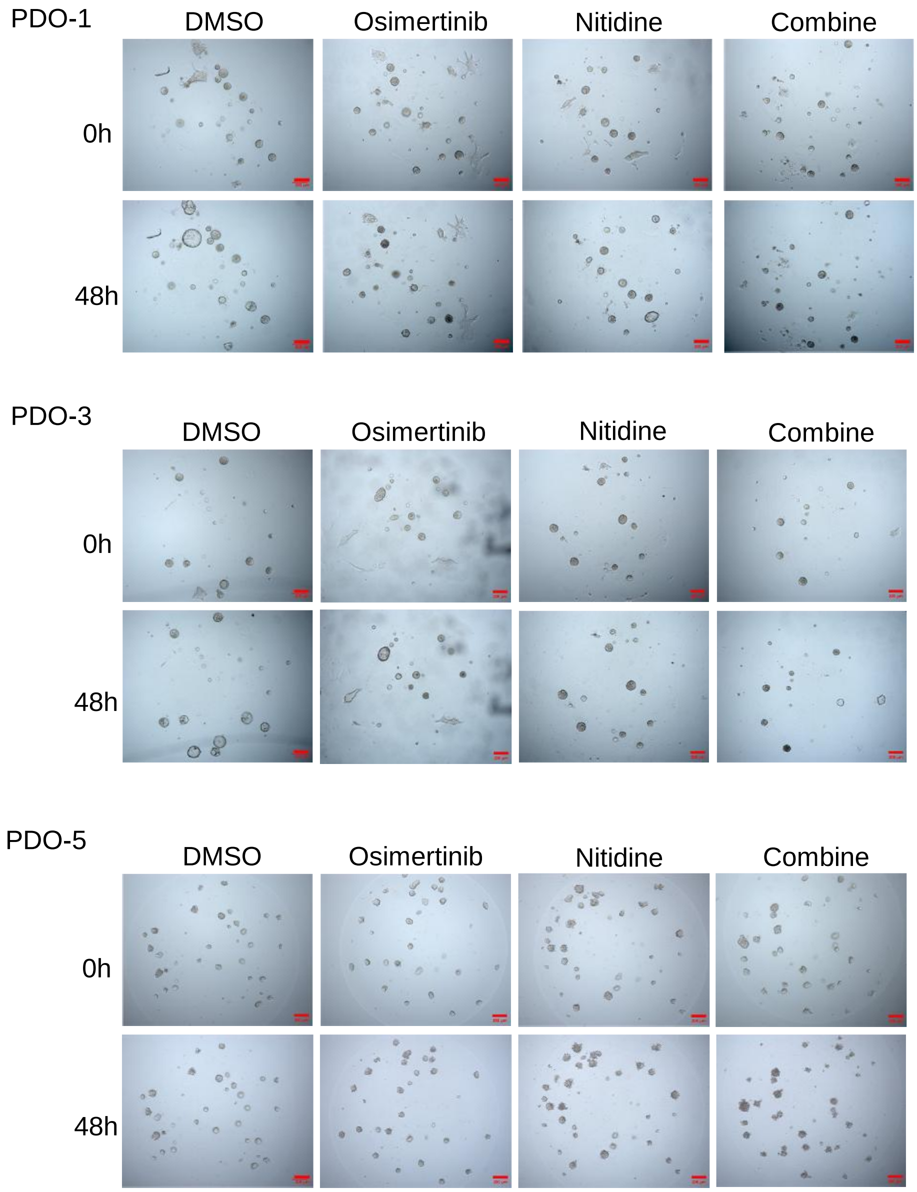

B

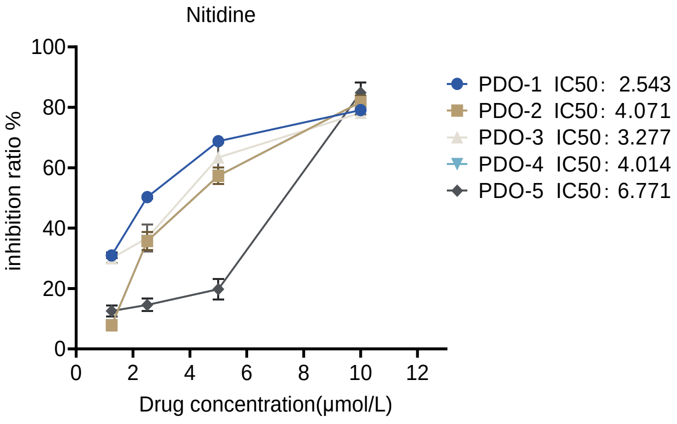

C

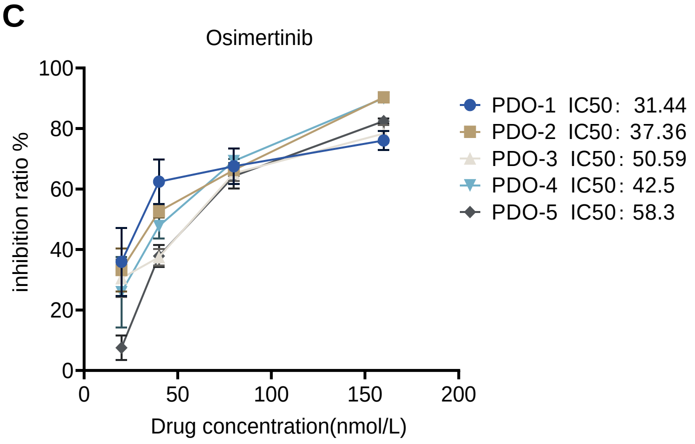

Figure S6

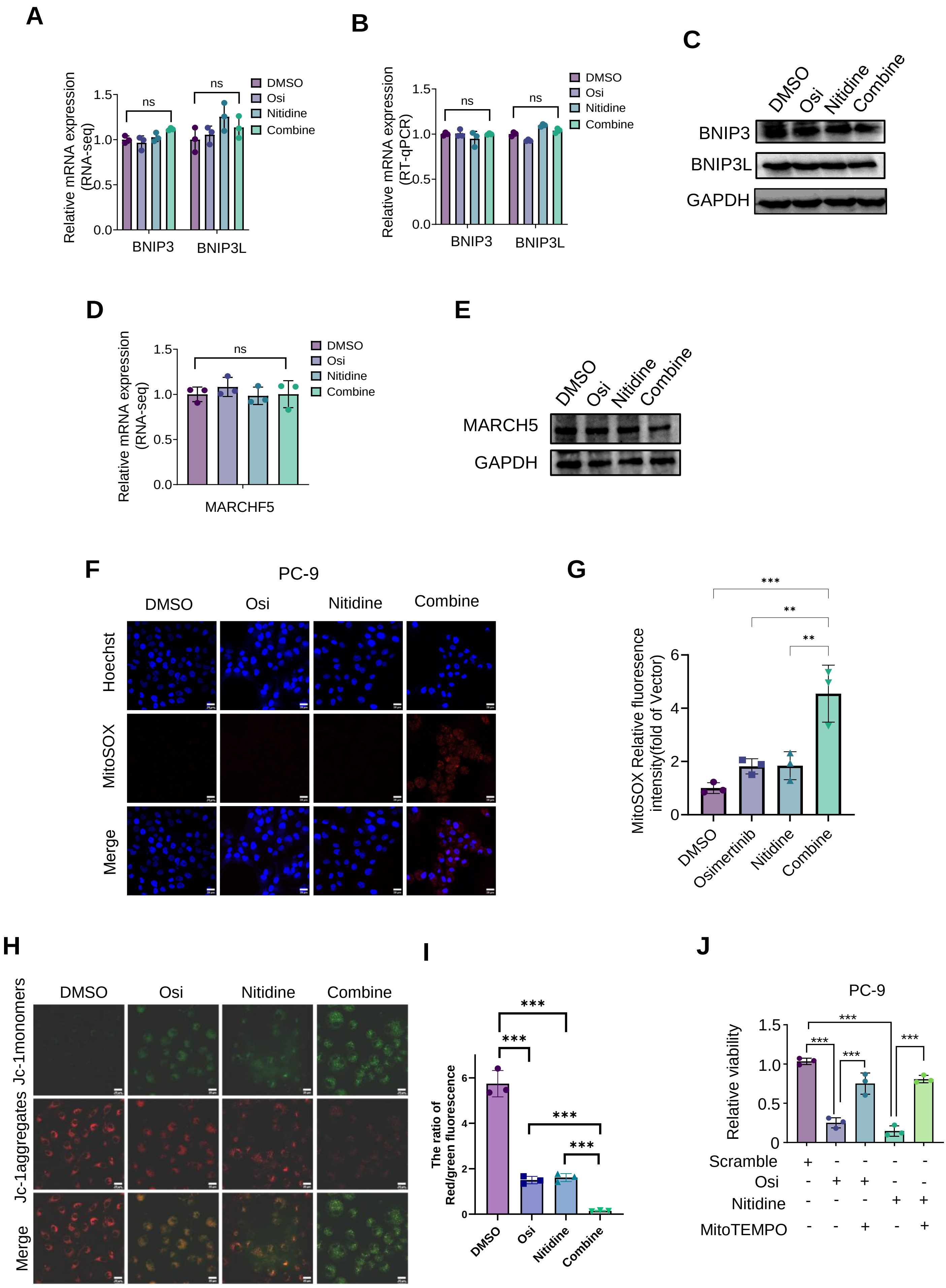

A
Relative mRNA expression (RNA-seq)
1.5
1.0
0.5
0.0
ns
ns
BNIP3
BNIP3L
DMSO
Osi
Nitidine
Combine
B
Relative mRNA expression (RT-qPCR)
C
DMSO
Osi
Nitidine
Combine
BNIP3
BNIP3L
GAPDH
D
MARCHF5
E
MARCH5
GAPDH
F
PC-9
Hoechst
MitoSOX
Merge
G
MitoSOX Relative fluoresence intensity(fold of Vector)
***
**
**
DMSO
Osimertinib
Nitidine
Combine
H
Jc-1monomers
Jc-1aggregates
Merge
I
The ratio of Red/green fluorescence
***
J
PC-9
Relative viability
Scramble
Osi
Nitidine
MitoTEMPO

Figure S7

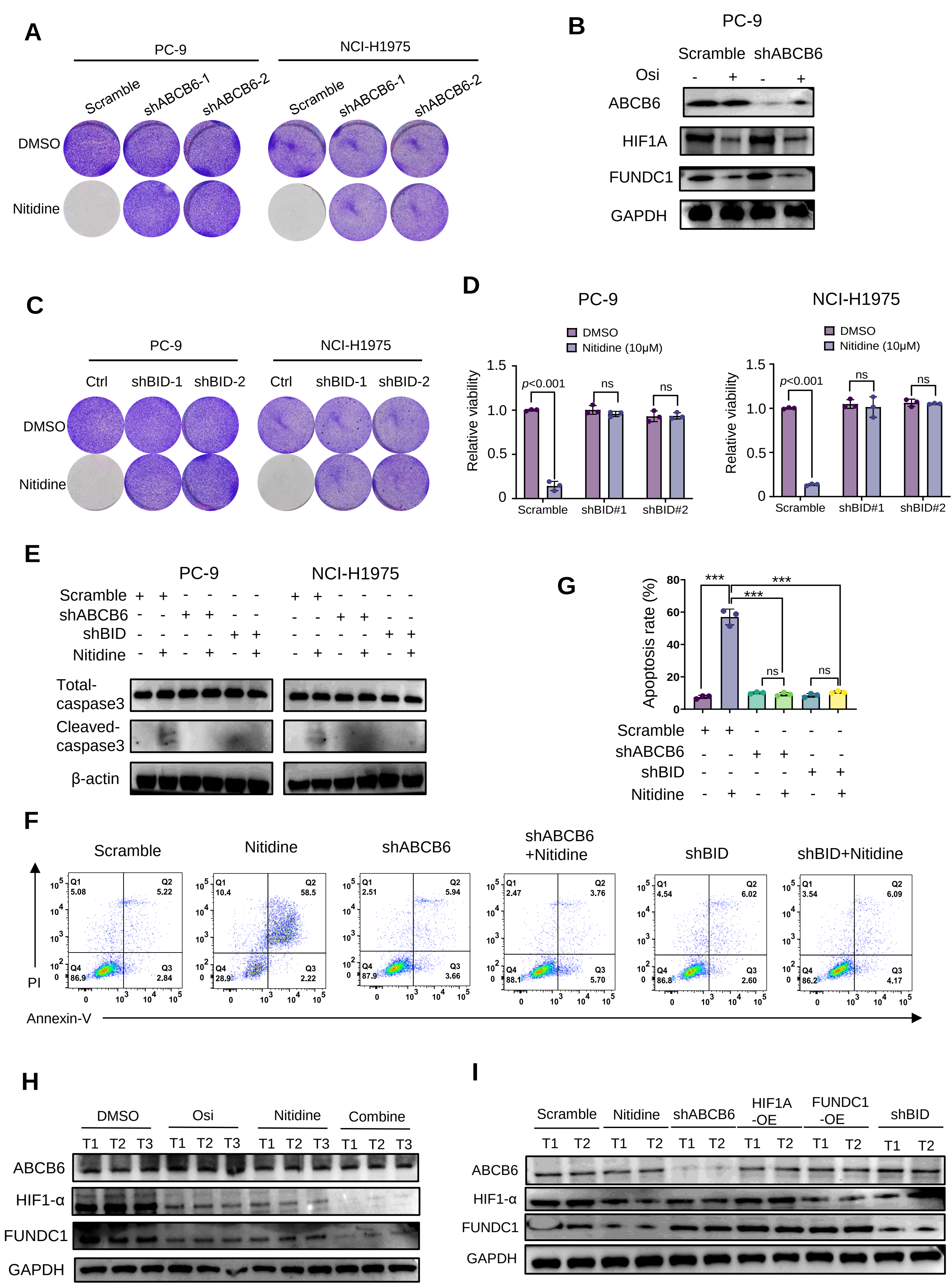

A
PC-9
NCI-H1975
Scramble
shABCB6-1
shABCB6-2
DMSO
Nitidine
B
PC-9
Scramble shABCB6
Osi - + - +
ABCB6
HIF1A
FUNDC1
GAPDH
C
PC-9
NCI-H1975
Ctrl shBID-1 shBID-2
D
PC-9
NCI-H1975
DMSO
Nitidine (10μM)
Relative viability
p<0.001
ns
Scramble
shBID#1
shBID#2
E
Scramble
shABCB6
shBID
Nitidine
Total-caspase3
Cleaved-caspase3
β-actin
G
Apoptosis rate (%)
***
ns
F
Scramble
Nitidine
shABCB6
shABCB6 +Nitidine
shBID
shBID+Nitidine
PI
Annexin-V
H
DMSO
Osi
Nitidine
Combine
T1 T2 T3
ABCB6
HIF1-α
FUNDC1
GAPDH
I
Scramble
Nitidine
shABCB6
HIF1A -OE
FUNDC1 -OE
shBID
T1 T2

Figure S8

A

Case	Sex	Age (years)	Stage/status at recurrence or progression	EGFR genotype / acquired alterations	Co-alterations (if available)
Patient 1	Female	61	Postoperative recurrence; pT2N0M0, IB stage	EGFR L858R	MET amplification
Patient 2	Female	71	Progression; cT3N1M1, IVA stage	EGFR exon 19 deletion; acquired EGFR T790M (re-biopsy) prior to osimertinib	Not available
Patient 3	Male	67	Progression; cT1N2M1, IVA stage	EGFR exon 19 deletion; Acquired EGFR T790M (re-biopsy) prior to osimertinib	TP53, RB1 mutation
Patient 4	Female	43	Progression; cT3N2M1, IVA stage	EGFR exon 19 deletion; acquired EGFR T790M (re-biopsy) prior to osimertinib	TP53 exon 8 missense; RB1 alteration
Patient 5	Male	75	Progression; cT4N3M0, IIIC stage	EGFR L858R	Not available
Patient 6	Female	48	Progression; cT4NxM1, IVA stage	EGFR L858R (exon 21); EGFR L833V (exon 21)	TP53 exon 7 missense; RB1 exon 1 frameshift
Patient 7	Male	58	Postoperative recurrence; pT2N1M0, IIB stage	EGFR exon 19 deletion	Not available
Patient 8	Female	40	Progression; cT3N1M1, IVA stage	EGFR exon 20 T790M	PD-L1 TPS 50%
Patient 9	Male	56	Progression; cT2N1M0, IIIB stage	EGFR L858R mutation	None
Patient 10	Female	61	Progression; cT2N2M1, IVA stage	EGFR L858R T790M mutation	PTEN exon 5 R130X

B

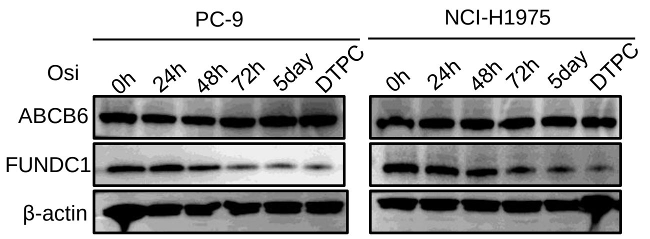

Table S1

Name	Formula	Mass (Da)	RT (min)	Library Score	Area	Ion mode
Xanthotoxol	C11H6O4	202.0266	0.65	100%	2017	Pos
sn-glycero-3-Phosphocholine	C8H20NO6P	257.1028	0.67	99%	1935	Pos
L-Carnitine	C7H15NO3	161.1052	0.69	92%	1104	Pos
Adenine	C5H5N5	135.0545	0.72	95%	5141	Pos
Taurine	C2H7NO3S	125.0147	0.74	100%	3340	Neg
Guanosine	C10H13N5O5	283.0917	0.75	100%	1328	Pos
D-Lactic acid	C3H6O3	90.0317	0.87	93%	4794	Neg
Mesaconic acid	C5H6O4	130.0266	1.83	96%	2188	Neg
Protocatechuic acid	C7H6O4	154.0266	2.55	98%	1166	Neg
2,2-Dimethylsuccinic acid	C6H10O4	146.0579	2.64	97%	1027	Neg
Phenylpropiolic acid	C9H6O2	146.0368	2.65	99%	8939	Neg
5-Hydroxymethylfurfural	C6H6O3	126.0317	2.77	83%	2085	Neg
7-Methoxycoumarin	C10H8O3	176.0473	3.03	84%	1436	Neg
Ureidosuccinic acid	C5H8N2O5	176.0433	3.03	84%	1322	Neg
Allantoic acid	C4H8N4O4	176.0546	3.03	84%	1368	Neg
6-Hydroxynicotinic acid	C6H5NO3	139.0269	3.06	75%	1464	Neg
Isoxanthopterin	C6H5N5O2	179.0443	3.36	84%	1094	Neg
Allantoin	C4H6N4O3	158.0440	3.51	90%	6870	Neg
L-Dihydroorotic acid	C5H6N2O4	158.0328	3.51	90%	4824	Neg
Suberic acid	C8H14O4	174.0892	3.98	97%	1237	Neg
Flavone	C15H10O2	222.0681	4.13	86%	18130	Neg
Isoscopoletin	C10H8O4	192.0423	4.25	82%	1136	Pos
Quinic acid	C7H12O6	192.0634	4.64	97%	7419	Neg
3-Butylidenephthalide	C12H12O2	188.0837	4.67	100%	50989	Neg
Vasicine	C11H12N2O	188.0950	4.67	100%	47167	Neg
Azelaic acid	C9H16O4	188.1049	4.68	100%	2620	Neg
Cotinine	C10H12N2O	176.0950	4.68	75%	4232	Pos
Prim-O-glucosylcimifugin	C22H28O11	468.1631	4.74	79%	6562	Neg
Isofraxidin	C11H10O5	222.0528	4.83	86%	1882	Neg
L-Cystathionine	C7H14N2O4S	222.0674	4.83	86%	16065	Neg
Guanosine monophosphate	C10H14N5O8P	363.0580	4.90	78%	2465	Neg
Camptothecin	C20H16N2O4	348.1110	4.90	66%	2363	Neg
Salidroside	C14H20O7	300.1209	4.96	80%	2372	Pos
Farrerol	C17H16O5	300.0998	4.96	72%	4913	Pos
Curculigoside	C22H26O11	466.1475	5.04	99%	11489	Neg
Sesamoside +HCOOH	C17H24O12.HCOOH	466.1323	5.11	76%	6422	Neg
Alpinetin	C16H14O4	270.0892	5.14	61%	2881	Neg
Artemisinin	C15H22O5	282.1467	5.48	81%	17049	Pos
L-Asparagine	C4H8N2O3	132.0535	5.62	91%	1454	Pos

Cinnamaldehyde	C9H8O	132.0575	5.62	91%	1415	Pos
5-Methoxytryptophan	C12H14N2O3	234.1004	5.76	94%	1488	Pos
4-Hydroxybenzoic acid	C7H6O3	138.0317	5.82	95%	7968	Pos
Atrolactic Acid	C9H10O3	166.0630	5.82	99%	4260	Neg
7-Methylxanthine	C6H6N4O2	166.0491	5.82	99%	90348	Neg
Ononin	C22H22O9	430.1264	6.08	82%	14118	Neg
7-O-Ethylmorroniside	C19H30O11	434.1788	6.47	84%	3715	Neg
Glycocholic acid	C26H43NO6	465.3091	6.53	88%	101935	Pos
Hypericin	C30H16O8	504.44	6.55	82%	15484	Pos
Sibirioside A	C21H28O12	472.1581	6.58	86%	8621	Neg
PicrosideII	C23H28O13	512.1530	6.70	72%	6869	Neg
Vitamin A acid	C20H28O2	300.2089	7.09	77%	29985	Pos
Patchouli alcohol (loss H20)	C15H24	204.1878	7.12	88%	41813	Pos
7-Demethylsuberosin	C14H14O3	230.0943	7.13	78%	1893	Pos
Carvacrol	C10H14O	150.1045	7.28	82%	8897	Pos
Curcumenol	C15H22O2	234.1620	7.93	95%	183897	Pos
Curdione	C15H24O2	236.1776	7.97	79%	67918	Pos
5-Methoxydimethyltryptamine	C13H18N2O	218.1419	8.51	88%	6705	Pos
Harmine	C13H12N2O	212.0950	8.71	96%	4176	Pos
Nitidine	C21H18NO4	348.1236	8.73	81%	12044	Neg
Pregnenolone	C21H32O2	316.2402	8.81	93%	3987	Neg
Spinosin	C28H32O15	608.1741	9.01	88%	12922	Neg
Pimelic acid	C7H12O4	160.0736	9.12	95%	5236	Pos
6-Methylcoumarin	C10H8O2	160.0524	9.12	95%	14140	Pos
Citramalic acid	C5H8O5	148.0372	9.12	72%	39857	Pos
Oxoadipic acid	C6H8O5	160.0372	9.12	95%	4651	Pos
Polygalic acid	C29H44O6	488.3138	9.13	100%	30466	Neg
Retinal	C20H28O	284.2140	9.43	92%	1834	Neg
4'-Hydroxyacetophenone	C8H8O2	136.0524	9.47	74%	3301	Pos
Aristolone	C15H22O	218.1671	9.53	72%	49998	Pos
Alantolactone	C15H20O2	232.1463	10.09	87%	31551	Pos
Bufalin	C24H34O4	386.2457	11.98	79%	18919	Pos
Verapamil	C27H38N2O4	454.2832	12.34	100%	1432	Neg
Muscone	C16H30O	238.2297	12.90	75%	5311	Pos

Table S2

Gene	Primer name	Sequence (5'-3')	Application
HIF1A	HIF1A-RT-F	TATGAGCCAGAAGAACTTTTAGGC	RT-qPCR
HIF1A	HIF1A-RT-R	CACCTCTTTTGGCAAGCATCCTG	RT-qPCR
ABCB6	ABCB6-RT-F	GTTCTTCAACGCCTGGTTTGGC	RT-qPCR
ABCB6	ABCB6-RT-R	AGCACGACGAAACTTGGTTCTCC	RT-qPCR
FUNDC1	FUNDC1-RT-F	AGACACCAGTGGTGGAATCGAG	RT-qPCR
FUNDC1	FUNDC1-RT-R	TCTGGAACAGAAATCCTGCACAC	RT-qPCR
BNIP3	BNIP3-RT-F	TCAGCATGAGGAACACGAGCGT	RT-qPCR
BNIP3	BNIP3-RT-R	GAGGTTGTCAGACGCCTTCCAA	RT-qPCR
NIX	NIX-RT-F	TGTGGAAATGCACACCAGCAGG	RT-qPCR
NIX	NIX-RT-R	CTACTGGACCAGTCTGATACCC	RT-qPCR
ACTB	ACTB-RT-F	CACCATTGGCAATGAGCGGTTC	RT-qPCR
ACTB	ACTB-RT-R	AGGTCTTTGCGGATGTCCACGT	RT-qPCR
RPL13A	RPL13A-F	CGCCCTACGACAAGAAAAAG	nuclear DNA reference for mtDNA/nDNA
RPL13A	RPL13A-R	CCGTAGCCTCATGAGCTGTT	nuclear DNA reference for mtDNA/nDNA
mtCO1	mtCO1-F	CAGGAGTAGGAGAGAGGGAGGTAAG	mtDNA/nDNA assay
mtCO1	mtCO1-R	TACCCATCATAATCGGAGGCTTTGG	mtDNA/nDNA assay
mtATP6	mtATP6-F	GAAGCGCCACCCTAGCAATA	mtDNA/nDNA assay
mtATP6	mtATP6-R	GCTTGGATTAAGGCGACAGC	mtDNA/nDNA assay
mtCYTB	mtCYTB-F	AGACAGTCCCACCCTCACAC	mtDNA/nDNA assay
mtCYTB	mtCYTB-R	GTTGTTTGATCCCGTTTCGT	mtDNA/nDNA assay
